# Genome-wide identification and characterization of *CONSTANS-like *gene family in radish (*Raphanus sativus*)

**DOI:** 10.1371/journal.pone.0204137

**Published:** 2018-09-24

**Authors:** Tianhua Hu, Qingzhen Wei, Wuhong Wang, Haijiao Hu, Weihai Mao, Qinmei Zhu, Chonglai Bao

**Affiliations:** Institute of Vegetable, Zhejiang Academy of Agricultural Sciences, Hangzhou, China; Chungnam National University, REPUBLIC OF KOREA

## Abstract

Floral induction that initiates bolting and flowering is crucial for reproductive fitness in radishes. *CONSTANS*-like (*CO*-like, *COL*) genes play an important role in the circadian clock, which ensures regular development through complicated time-keeping mechanisms. However, the specific biological and functional roles of each *COL* transcription factor gene in the radish remain unknown. In this study, we performed a genome-wide identification of *COL* genes in the radish genome of three cultivars including ‘Aokubi’, ‘kazusa’ and ‘WK10039’, and we analyzed their exon-intron structure, gene phylogeny and synteny, and expression levels in different tissues. The bioinformatics analysis identified 20 *COL* transcription factors in the radish genome, which were divided into three subgroups (Group I to Group III). *RsaCOL-09* and *RsaCOL-12* might be tandem duplicated genes, whereas the others may have resulted from segmental duplication. The Ka/Ks ratio indicated that all the *COL* genes in radish, *Arabidopsis*, *Brassica rapa*, *Brassica oleracea*, *Capsella rubella* and rice were under purifying selection. We identified 6 orthologous and 19 co-orthologous *COL* gene pairs between the radish and *Arabidopsis*, and we constructed an interaction network among these gene pairs. The expression values for each *COL* gene during vegetable and flower development showed that the majority of Group I members had similar expression patterns. In general, the expression of radish *COL* genes in Groups I and III decreased during development, whereas the expression of radish *COL* genes in Group II first increased and then decreased. Substantial numbers of radish *COL* genes were differentially expressed after vernalization treatment. The expression levels of *RsaCOL-02* and *RsaCOL-04* were significantly increased during vernalization treatment, while the expression of *RsaCOL-10* was significantly decreased. These outcomes provide insights for improving the genetic control of bolting and flowering in radish and other root vegetable crops, and they facilitate genetic improvements to radish yields and quality.

## Introduction

The transition from vegetative development to bolting and flowering is critical for reproductive success in the plant life cycle. The initiation of flowering is controlled by complex genetic networks and could be effected by diverse plant hormones and environmental factors such as light, temperature, and length of day [1–3]. In *Arabidopsis thaliana*, approximately 180 genes participate in flowering-time control, and they are involved in six interactive regulatory pathways including the vernalization pathway, autonomous pathway, photoperiod pathway, gibberellin (GA) pathway, ambient temperature pathway, and age pathway [1,3,4]. These genes interact within different pathways to ensure that flowering occurs under appropriate conditions. For example, the primary flowering repressor *FLOWERING LOCUS C* (*FLC*) encodes a MADS-box transcription factor, and it integrates both the autonomous and the vernalization pathways [5]. Other flowering pathway integrators such as *FLOWERING LOCUS T* (*FT*), *SUPPRESSOR OF OVEREXPRESSION OF CONSTANS 1* (*SOC1*) and *LEAFY* (*LFY*) were also confirmed as convergence points for different flowering pathways [6–8].

*CONSTANS*-like (*CO*-like, *COL*) genes play a vital role in the regulation of plant flowering through the photoperiod pathway, integrating the circadian clock, light signals and meristem genes identified as flowering time controls [9–11]. As a phloem-specific transcription activator of *FT*, *CO* promotes flowering by up-regulating the transcription of the *FT* and *TWIN SISTER OF FT* (*TSF*) genes [12]. At the posttranscriptional level, *CO* is degraded by the ubiquitin ligase CONSTITUTIVE PHOTOMORPHOGENIC 1 (*COP1*) in the dark, and, in the morning, it is degraded by a pathway activated by the photoreceptor *PHYTOCHROME B* (*PHYB*). These transcriptional and posttranscriptional regulations ensure that *CO* activates *FT* and *TSF* transcription only during long days. *CO* belongs to an *Arabidopsis* gene family containing 16 other genes that encode putative transcription factors [13]. Structurally, typical *COL* genes contain two conserved domains, a plant-specific C-terminal CCT (also termed CO, *CO*-like, TOC1) domain and an N-terminal zinc finger B-box domain, which is also found in animals [14]. The *COL* genes belong to a larger transcription factor gene family named the B-BOX (BBX) family, which can be divided into five groups based on the presence of one or two B-BOX motifs and the presence or absence of the CCT domain [15]. Previous studies showed that most *COL* genes containing a CCT domain are involved in controlling the flowering time in some plant species [16,17].

The availability of the *Arabidopsis* genome sequence and annotation provides a useful tool for a comparative analysis of *COL* transcriptional regulators. In addition, many tools and databases such as the PlantTFDB database (version 3.0, http://planttfdb.cbi.pku.edu.cn) were developed for identifying, clustering, aligning and functionally analyzing plant transcription factors [18]. The *CO*-like genes in *Arabidopsis* are classified into three major groups; group I included *AtCO* and *AtCOL1* to *AtCOL5* with two B-boxes; group II contained *AtCOL6* to *AtCOL8* and *AtCOL16* with one B-box; and group III consisted of COL9-COL15 with one B-box and another diverged zinc finger domain [13,19]. An analysis identified 17 *COL* genes in *Oryza sativa* that belonged to 30 rice *BBX* genes, and they were divided into four phylogenetic groups [17]. Genome-wide analyses have also been performed for *COL* transcription factors in the Brassicaceae family. Song *et al*. (2015) found 25 *Brassica rapa COL* genes, and they investigated the evolutionary pattern of *COL* genes in 34 Angiospermae (27 eudicots, six monocots and one basal angiosperm), three Gymnospermae, one Pteridophyta, one Bryophyta and six Chlorophyta species [20].

The radish (*Raphanus sativus* L., 2n = 2x = 18) is an annual diploid species of the Brassicaceae family, and it is also an economically important vegetable crop that is produced worldwide. The primary edible part of the radish is the tuberous root, and it contains various nutrients and medicinal compounds [21]. The optimum timing of bolting and flowering are vital for economic organ production and for preventing premature bolting and flowering in radishes. Recently, one draft genome assembly of the radish *Raphanus sativus* var. *hortensis* was published, resulting in a total of 383 Mb of sequences and 54,357 genes [22]. In 2016, another genome assembly of the radish *R*. *sativus* cv. WK10039 was published, which containing 344.0 Mb of sequences could be integrated into nine chromosome pseudomolecules out of 426.2 Mb total sequences. In total, 46,514 protein-coding genes were predicted and annotated [23]. The transcriptomes of various tissues in radish including the roots and leaves, have been assembled and analyzed [24–27]. These databases provide powerful tools for the genome-wide characterization of bolting and flowering genes in radish. Previous studies have reported a list of functional genes and microRNAs (miRNAs) related to radish bolting and flowering based on transcriptomic sequencing, expression profiling and transgenic approaches [2,28,29]. However, the specific biological and functional roles of the *CO*-like transcription factor genes in radish remain unknown.

This study is the first report on the genome-wide identification of *CO*-like genes in the radish. In the present study, we identified the *COL* genes in the radish genome and classified them into three groups based on the analysis of their exon-intron structure, gene phylogeny and synteny, as well as their expression in specific tissues. The results provide insights into the genetic networks regulating bolting and flowering in radish and other vegetable crops in Brassicaceae, and they will also facilitate the genetic improvement of radish yield, nutritional value and commercial quality.

## Materials and methods

### Sequence retrieval and *COL* members identification in radish

The genome, genes and corresponding protein sequences of the radish were downloaded from the NODAI Radish Genome Database (http://www.nodai-genome-d.org, cultivar ‘Aokubi’), Radish Genome Database (http://radish-genome.org) and Raphanus sativus Genome Database (http://radish.kazusa.or.jp/, cultivar ‘kazusa’). The Markov models of two identical domains for the *CO*-like transcription factors, PF06203.13 (CCT) and PF00643.23 (zinc finger B-box), were downloaded from the Pfam database (http://pfam.xfam.org/) [30,31]. All radish proteins were aligned with these two Markov models using the HMMER program with a cut-off E-value of 1e^-4^ separately. Only the proteins that were aligned with both models were selected as *CO*-like protein candidates. The *COL* genes that were located at a distance of 10 kb on the same chromosome or scaffold were considered tandemly duplicated genes.

### *COL* genes identification in representative plants

The genome and annotated proteins of 19 representative species in the primary lineages of the plant kingdom were also collected. The annotated proteins of algae (*Chlamydomonas reinhardtii*), *Coccomyxa subellipsoidea*, *Dunaliella salina*, moss (*Physcomitrella patens*), lycophyte (*Selaginella moellendorffii*), *Amborella trichopoda*, *Aquilegia coerulea*, *Arabidopsis thaliana*, *Capsella rubella*, *Eutrema salsugineum*, grape (*Vitis vinifera*), poplar (*Populus trichocarpa*), tomato (*Solanum lycopersicum*), maize (*Zea mays*) and rice (*Oryza sativa*) were downloaded from the Pfam database (v11) [32]. The genomic information on the gymnosperm plant Norway spruce (*Picea abies*) [33] was collected from the Congenie Website (http://congenie.org/). The sequences of the sacred lotus genes and proteins were downloaded from Lotus-DB (http://lotus-db.wbgcas.cn/, v1.0) [34]. The proteins of *Brassica rapa* were obtained from the BRAD (*Brassica* database, http://brassicadb.org/brad/), those of *B*. *napus* from the GENOSCOPE database (http://www.genoscope. cns.fr/brassicanapus/data/) and those of *B*. *oleracea* from BolBase (http://www.ocri-genomics. org/bolbase/, v1.0). The candidate proteins that only contain the fragmental CCT or zinc finger B-box domains were eliminated manually. Protparam (http://web.expasy.org/protparam/) [35] was employed in the physical and chemical characteristic analysis of the COL proteins in those analyzed species, including the molecular weight, theoretical isoelectric point (pI), atomic composition formula, instability index, aliphatic index and grand average of hydropathicity (GRAVY).

### Phylogenetic analysis of the *COL* genes

The collection of protein sequences from multiple species was used for a phylogenetic analysis in *Planta*. First, a pairwise alignment and a multiple alignment were performed using the ClustalX2 program with the Gonnet protein weight matrix [36], and then a maximum likelihood phylogenetic tree was built with the MEGA program (v6.06) using the Jones-Taylor-Thornton (JTT) model with 1000 bootstrap replicates using the full CDS sequence for a partial 70% length [37]. Uniform rates and homogeneous lineages were adopted, and the partial deletion with a site coverage cutoff of 70% were used for gaps/missing data treatment. The frequency of each divergent branch was displayed when it was higher than 50%. The figure was beautified with information from the group using Adobe Illustrator software.

### Gene structure and motif analysis

The gene structure was analyzed using the Gene Structure Display Server tool (http://gsds.cbi.pku.edu.cn/, v2.0) [38]. MEME software (http://meme.nbcr.net/meme/, v4.12.0) was used to search for motifs among the proteins [39]. The searching motif window length was set from 10 bp to 100 bp. Only widely distributed motifs that occurred in at least 3 protein sequences were retained. These motifs were drawn in two separate figures, in accordance with the phylogenetic trees. The top 10 motifs with the lowest E-values were reported, which were displayed according to a pattern in which the more statistically significant (lower E-value) motifs came first.

### Identification of orthologous and paralogous genes

OrthoMCL software (v2.0.3) [40] was employed in searching for orthologous, co-orthologous and paralogous genes in radish, *Arabidopsis*, *Brassica rapa*, *Brassica oleracea*, *Capsella rubella* and rice using entire COL protein sequences. The E-value cut-off of an all-against-all BLASTP alignment process was set at 1e^-5^, and the alignment with a match cut-off value lower than 50 was eliminated. To evaluate the divergence of duplicated radish COL genes, the synonymous rate (Ks), nonsynonymous rate (Ka), and evolutionary constraint (Ka/Ks) between paralogous pairs of genes were calculated with the KaKs_calculator tool and paraAT software using the method developed by Nei and Gojobori (http://cbb.big.ac.cn/software). The divergence time was calculated for homologous pairs among five dicotyledonous plants using the formula T  =  Ks/2R, where R is the rate of divergence for nuclear genes from plants, and it was considered equal to 1.5 × 10^−8^ synonymous substitutions per site per year for dicotyledonous plants [41]. The selected homologous pairs of four species including radish, *Arabidopsis*, *Brassica rapa*, and rice were gathered and displayed using Cytoscape software (http://www.cytoscape.org, v2.8.3) [42].

### Gene expression analysis in radish tissues and cytoplasmic male sterility and in response to vernalization

The gene expression data of the *COL* genes were gathered from the NODAI genome database, which was used in a previous report [22]. The RNA-seq data from the 7 d root, 7 d leaf, 14 d root, 14 d leaf, 20 d root, 20 d leaf, 40 d cortex, 40 d cambium, 40 d xylem, 40 d root tip, 40 d leaf, 60 d cortex, 60 d cambium, 60 d xylem, 60 d root tip, 60 d leaf, 90 d cortex, 90 d cambium, 90 d xylem, 90 d root tip, and 90 d leaf of Japanese radish cultivar ‘Aokubi’ were used. The gene expression profile in each sample was standardized using the RPKM (reads per kilobase per million measure) method. The expression profile of the *COL* genes from each sample was analyzed using the HemI program (http://hemi.biocuckoo.org/) with the average hierarchical clustering method after being normalized by a logarithmic base (log2). The hierarchy was obtained by clustering the gene expression in both the horizontal axis and the vertical axis using a Pearson distance similarity metric. A discrete bar containing 21 colors was used to represent the gene expression values.

Furthermore, high-throughput RNA sequencing was performed to characterize the transcriptome of radish buds with a length that was approximately 1.5 mm from two CMS lines (HYBP-B and YH-B) possessing the CMS-inducing orf138 gene and corresponding to near-isogenic maintainer lines (HYBP-A and YH-A) [43]. The sequencing data of 4 radish lines were downloaded from the NCBI SRA database under bioproject PRJNA273265, with two replicates sequenced for each line. The TopHat program (https://ccb.jhu.edu/software/tophat/ index.shtml, v2.1.0) was used to map the reads to the NODAI radish genome; the expression profile of all the spinach genes was then obtained with the FPKM (Fragments Per Kilobase of exon per million fragments Mapped) value using Cufflinks software (http://cole-trapnell-lab.github.io/cufflinks, v2.2.1) under the guidance of annotated gene models with a GFF file. The radish COL gene expression profiles from each sample were analyzed using the HemI program.

The gene expression of the radish seedlings at three different time points during vernalization in another report was also used in this study. The samples were labeled RT1, RT2, and RT3 for the room temperature treatment (RT), VE1, VE2, and VE3 for the early stage of vernalization (VE), and VL1, VL2, and VL3 for the late stage of vernalization (VL) [44]. The gene expression value obtained by the FPKM method was then drawn using the HemI program.

### Prediction of the regulating network of radish *COL* genes

The interaction network of radish *COL* genes was constructed using information in *Arabidopsis* Interactions Viewer (http://bar.utoronto.ca/interactions/cgi-bin/arabidopsis_interactions_viewer.cgi) to build an *Arabidopsis COL* regulating network, and then each *Arabidopsis* gene was replaced by the corresponding radish orthologs and co-orthologs. This approach is based on the theory that homologs are structurally and functionally similar. In addition, the gene expression of radish organs during different developmental stages were also used to calculate the Pearson correlation coefficient. First, the radish genes with low expression, including the highest expression lower than 2 or an accumulated expression lower than 5 in 21 sequencing samples, were eliminated. Second, the Pearson correlation coefficient was calculated for any combination of two radish gene expression values using R language. Finally, the gene expression values of the radish COL genes were only retained if the P-value was lower than 0.1 and if the absolute Pearson correlation coefficient (PCC) value was higher than 0.9. WEGO software (http://wego.genomics.org.cn/) was then used to plot a GO functional figure for these genes with GO term hits to view the distribution of gene functions [45].

## Results and discussion

### Genome-wide identification of *COL* genes in radish

The bioinformatics analysis identified 20 *COL* transcription factors in the radish genome among 54,357 coding genes. All of the radish *COL* genes were well conserved, and they were designated ‘*RsaCOL*’ with a serial number and sorted by the E-value of the CCT domain (S1 Table). The molecular weights of the COL proteins in the radishes ranged from 33134.20 to 46404.79, which was very similar to those in *Arabidopsis* (Table 1). The theoretical pI of the COL proteins in radish varied from 5.09 to 7.57, including one alkaline protein and 19 weakly acidic proteins. In contrast, all 17 COL proteins in *Arabidopsis* were acidic. In addition, the number of acidic proteins in other plants was less than 3. The aliphatic index of COL proteins ranged from 48.46 to 71.21, and the GRAVY index ranged from -0.704 to -0.318. The position of *COL* genes *RsaCOL-09* and *RsaCOL-12* were Rs_scaf1+: 669331, 670395 and Rs_scaf1+: 673375, 674690, which was located in close proximity. This finding indicated that these genes might be tandemly duplicated, whereas the others may have resulted from segmental duplication.

Several research centers have been interested in the whole sequencing of the radish genome and transcriptome, resulting in several versions of the radish genome and genetic information [22,46,47]. Till now, only one radish genome assembly contained gene location information in chromosome level, whereas other assemblies in scaffold level. In the present study, we primarily used the radish genome assembly in the NODAI Radish Genome Database to identify the *COL* genes in radish. In addition, we also ran HMMER software on other radish genome versions for a better comparison. Almost every *COL* gene in the ‘Aokubi daikon’ cultivar were matched with the corresponding *COL* gene in the ‘kazusa’ cultivar, except *RsaCOL*-20 (S2 Table). A total of 20 radish *COL* genes were also searched in the cultivar ‘WK10039’ (S2 Table), and their location in radish chromosomes were shown in S1 Fig. The availability of genome sequences from various species accelerates the genome-wide identification of gene families in plants. In radish, entire *MADS-box* genes and *WRKY* genes have been identified through genome-wide analysis [48,49]. The identification of *RsaCOL* genes in the present study provides additional knowledge about the gene family in radish and a foundation for further investigating the flowering regulatory networks in radish and other Brassicaceae vegetables.

**Table 1:** Classification and chemical characterization of radish *COL* genes.

ID	Gene	CDS Length	Whole Gene Length	Protein Length	Molecular Weight	pI	Formula	Instability index		Aliphatic index	GRAVY
RsaCOL-01	RSG31377	1206	1448	401	45258.99	5.80	C_1949_H_3080_N_574_O_615_S_27_	50.10	unstable	61.10	-0.711
RsaCOL-02	RSG42550	1209	1439	402	45545.01	5.43	C_1963_H_3086_N_574_O_628_S_24_	56.76	unstable	62.59	-0.755
RsaCOL-03	RSG21446	1041	1134	346	37997.54	6.08	C_1659_H_2573_N_471_O_521_S_17_	46.24	unstable	67.98	-0.316
RsaCOL-04	RSG07010	1245	1503	414	46404.79	5.19	C_2005_H_3147_N_579_O_648_S_21_	52.75	unstable	65.97	-0.711
RsaCOL-05	RSG35888	1029	1139	342	37407.90	5.59	C_1641_H_2542_N_450_O_520_S_16_	49.33	unstable	66.78	-0.248
RsaCOL-06	RSG11754	1047	1616	348	37611.95	6.02	C_1620_H_2534_N_474_O_522_S_19_	40.38	unstable	61.41	-0.451
RsaCOL-07	RSG34470	1059	1575	352	38036.25	5.68	C_1639_H_2554_N_474_O_535_S_18_	35.84	stable	61.28	-0.464
RsaCOL-08	RSG03422	1275	1604	424	47004.50	5.77	C_2001_H_3184_N_594_O_661_S_27_	44.00	unstable	60.02	-0.711
RsaCOL-09	RSG01422	1065	1065	354	39437.70	6.79	C_1674_H_2630_N_510_O_552_S_22_	60.02	unstable	57.34	-0.720
RsaCOL-10	RSG31262	924	1000	307	33134.20	6.60	C_1436_H_2271_N_419_O_455_S_14_	46.79	unstable	71.21	-0.318
RsaCOL-11	RSG03335	1113	1452	370	41063.03	6.36	C_1766_H_2803_N_521_O_568_S_20_	49.30	unstable	49.30	-0.696
RsaCOL-12	RSG01423	1128	1316	375	42777.35	7.57	C_1820_H_2846_N_562_O_592_S_22_	60.57	unstable	52.80	-0.970
RsaCOL-13	RSG01107	1092	1641	363	40845.23	6.57	C_1736_H_2748_N_526_O_578_S_19_	63.36	unstable	61.24	-0.825
RsaCOL-14	RSG23074	1134	1403	377	41112.13	5.47	C_1722_H_2677_N_521_O_592_S_30_	60.69	unstable	48.46	-0.704
RsaCOL-15	RSG09942	1062	1588	353	38890.86	5.75	C_1639_H_2544_N_490_O_555_S_28_	49.65	unstable	51.98	-0.663
RsaCOL-16	RSG40602	1161	1417	386	43064.44	5.74	C_1863_H_2920_N_522_O_598_S_27_	58.52	unstable	70.70	-0.541
RsaCOL-17	RSG03894	1074	1234	357	39387.21	5.09	C_1651_H_2564_N_492_O_572_S_29_	60.82	unstable	49.75	-0.746
RsaCOL-18	RSG13348	1050	1411	349	38859.07	5.71	C_1625_H_2552_N_490_O_553_S_33_	57.97	unstable	48.97	-0.705
RsaCOL-19	RSG14263	1203	1377	400	44361.63	5.91	C_1895_H_2964_N_552_O_616_S_32_	51.26	unstable	59.97	-0.508
RsaCOL-20	RSG28563	1002	2399	333	38743.45	6.13	C_1677_H_2636_N_502_O_523_S_17_	51.79	unstable	62.31	-0.925

### Phylogenetic relationship and evolutionary divergence of radish COL proteins

To provide insight into the evolution of *COL* genes in different species of the plant kingdom, we performed a comparative analysis using the genes from a total of 71 whole-genome-sequenced plant species. These species covered the primary lineages in *Planta*, including 7 algae, one lycophyte, one gymnosperm, a monocot, Eusasterids II, Euasterids I, Eurosids I, Eurosids II, Malpighiales, and some representative species, including Amborella, columbine, and sacred lotus.

**Fig 1:**
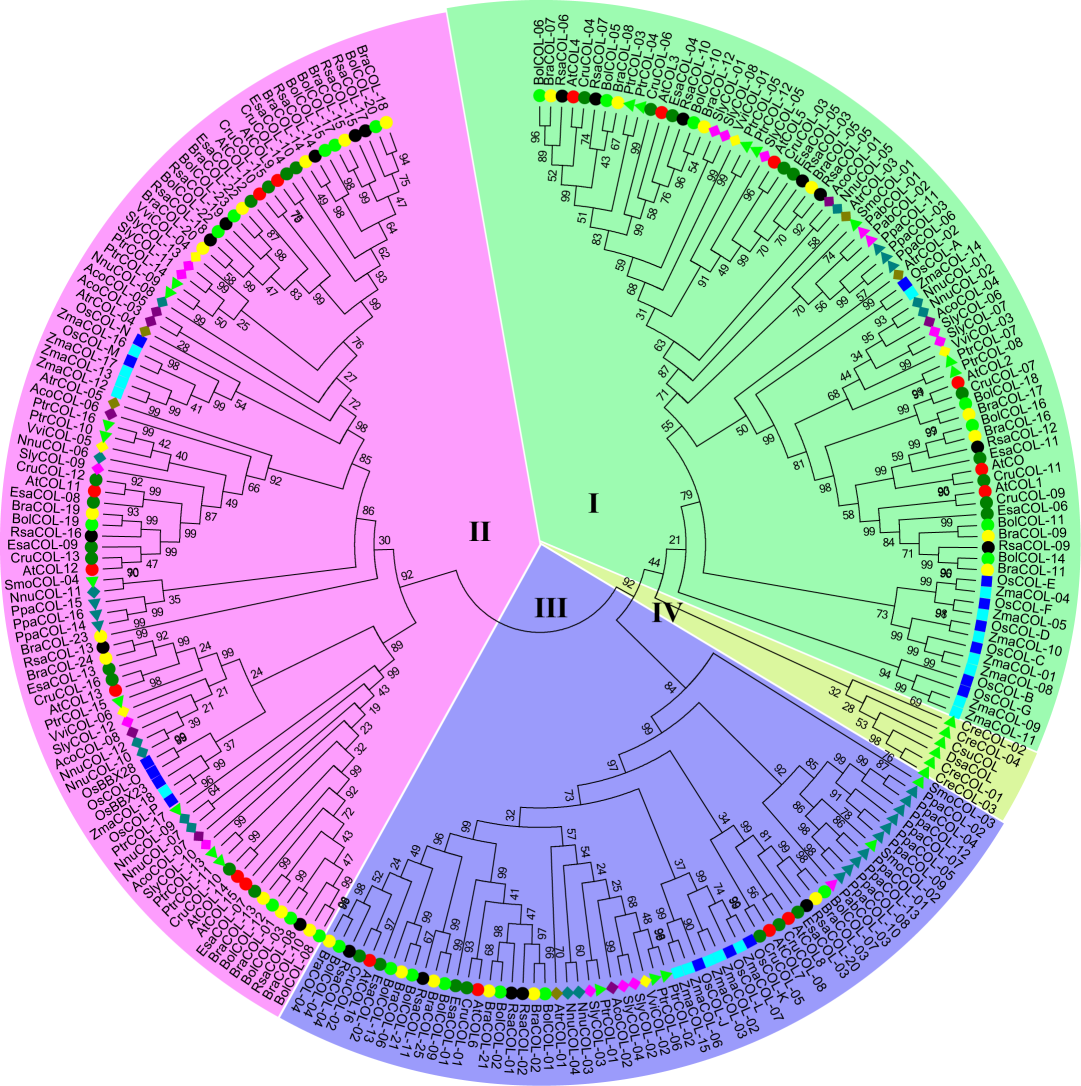
Phylogenetic tree of the COL proteins from 16 plant species. The phylogenetic tree was constructed based on the 90% shared amino acid sites using the maximum likelihood method. The abbreviations represent the species as follows: *At*, *Arabidopsis thaliana*; *Atr*, *Amborella trichopoda*; *Bra*, *Brassica rapa*; *Bol*, *Brassica oleracea*; *Cru*, *Capsella rubella*; *Esa*, *Eutrema salsugineum*; *Nnu*, *Nelumbo nucifera*; *Pab*, *Picea abies*; *Ppa*, *Physcomitrella patens*; *Ptr*, *Populus trichocarpa*; *Rsa*, *Raphanus sativus*; *Sly*, *Solanum lycopersicum*; *Sme*, *Selaginella moellendorffii; Tca*, *Theobroma cacao*; *Vvi*, *Vitis vinifera*; *Os*, *Oryza sativa*; and *Zma*, *Zea mays*.

We identified 5 *A*. *trichopoda COL* genes, 8 *A*. *coerulea COL* genes, 12 sacred lotus *COL* genes, 16 rice *COL* genes, 3 Norway spruce *COL* genes, 17 Populus *COL* genes, 6 grape *COL* genes and 18 maize *COL* genes (S3 Table). *C*. *subellipsoidea* and *D*. *salina* had the lowest number of *COL* genes, at one gene for each species. *C*. *reinhardtii* and *S*. *moellendorffii* had 4 *COL* genes each. The number of COL genes in *E*. *salsugineum* and tomato were also the same (13 *COL* genes for each species), whereas in the case of *C*. *rubella* and *P*. *patens*, the number of *COL* genes was 16 *COL* genes for each species. A previous report showed that *P*. *patens* contained 17 *COL* genes [50], whereas only 16 COL genes were identified in this report. Compared with other genera in Brassicaceae, those two *Brassica* species contained large numbers of *COL* genes, specifically, 23 genes in *B*. *oleracea* and 25 genes in *B*. *rapa*. This result was consistent with a previous report, and it was likely due to a genome triplication event [20]. To gain insight into the evolution of *COL* genes, we constructed a phylogenetic tree to characterize the families of *COL* genes (Fig 1). The phylogenetic tree was generally consistent with previous reports [19,20].

A previous analysis of *CO*-like genes in *Arabidopsis* classified the family into three broad groups based on the number and divergence of the B-box zinc finger domain [13]. In another study, the genes were referred to as Group I to III genes [19]. Song *et al*. classified the CO-like genes in *A*. *trichopoda*, *P*. *taeda*, *P*. *sitchensis*, *P*. *abies*, *S*. *moellendorffii* and *P*. *patens* into groups A to C according to the distribution of the B-box and zinc finger domains [20]. We grouped the *COL* genes into Groups I to III; the Group I members had two zinc finger B-boxes, whereas only one B-box was present in Groups II and III. Group III members had an additional diverged zinc finger.

In the present study, the *COL* genes in alga were grouped into a diverged branch; thus, the branches that contained *C*. *reinhardtii*, *C*. *subellipsoidea* and *D*. *salina* were named Group IV. The amount of a large number of transcription factor families in *P*. *abies* was several fold higher than that in *Arabidopsis* or *Physcomitrella*, which could be due to the polyploidy and complex nature of the *P*. *abies* genome. However, only two *COL* genes in Group I and one *COL* gene in Group III were identified. Similarly, one *COL* gene in Group I, one *COL* gene in Group II and two *COL* genes in Group III were identified in *S*. *moellendorffii*. Three, three and ten *COL* genes in *P*. *patens* were classified into Groups I, II and III, respectively. These genes were grouped tightly, indicating that these four clades were relatively more original than the other clades. The *COL* genes in monocots were closely grouped, and the rice *COL* genes had similar *COL* genes corresponding to maize *COL* genes. For example, every rice *COL* gene had a corresponding gene in maize in Group I, whereas in Group II, *OsCOL*-K, *OsCOL*-L and *OsCOL*-J had one, two and two corresponding *COL* genes in maize, respectively. In Group II, *OsCOL*-N had one corresponding gene in maize, and *OsCOL*-M had three corresponding genes in a branch, whereas four rice *COL* genes (*OsBBX28*, *OsCOL-O*, *OsBBX23* and *OsCOL-O*) corresponded to one maize gene. Genome duplication was also found in the Brassicaceae family. In several branches, one or two radish *COL* genes corresponded to the *Arabidopsis COL* gene. The phylogenetic tree showed the possibility of a *COL* gene that evolved divergently before the origin of *Lycopodium*, and it divided into three groups and evolved independently.

### Gene structure of *RsaCOL* genes

**Fig 2:**
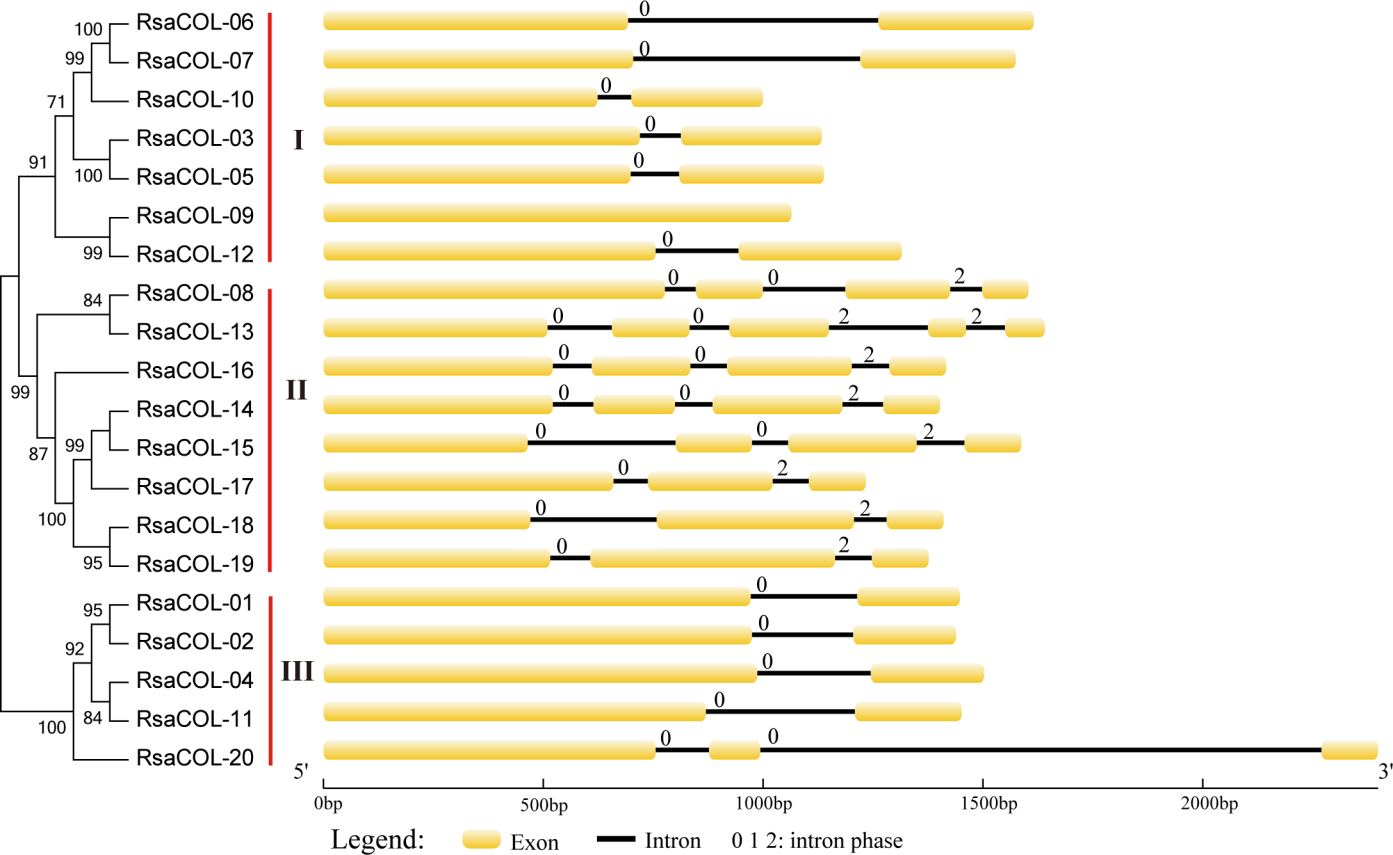
Gene structure of the radish *COL* genes. Red boxes represent exons, and black and blue lines represent introns and UTRs, respectively. The lengths of the exons and introns were drawn according to the lengths of the sequences.

To compare the radish *COL* genes, their exon-intron structures were predicted, and the results are shown in Fig 2. The phylogenetic trees of the *RsaCOL* genes were constructed by Maximum-Likelihood method with 1000 bootstraps using full CDS sequences. The *COL* genes in radish were divided into 3 groups, Group I, Group II and Group III, which is similar to the grouping in *Arabidopsis* and rice [19]. In general, genes in the same group have similar numbers of exons and introns and even intron phases, indicating that these genes shared a conserved splicing pattern. The *RsaCOL* genes in Group I contained two exons, except for *RsaCOL-12*, which contained one exon. In Group II, five *RsaCOL* genes contained four exons, whereas three *RsaCOL* genes contained three exons. Only *RsaCOL-20* contained two introns, whereas the other members in Group III contained one intron. The intron phase of all the intron-containing *RsaCOL* genes in Group I and Group III were 0, whereas the first intron phase and the last intron phase of all the intron-containing *RsaCOL* genes in Group II were 0 and 2, respectively. The length of the last intron in *RsaCOL-20* was over 1000 bp, while the other introns in all the *RsaCOL* genes were short. Because most of the genes shared conserved splicing patterns, we hypothesized that the intron increased event that occurred in *RsaCOL-20* might have resulted from a fragment insert based on the sequence analysis. In contrast, the intron loss event that occurred in *RsaCOL-09* resulted from a fragment deletion, and it contained a full intron and parts of exons.

### Conserved motifs located in *RsaCOL* protein sequences

**Fig 3:**
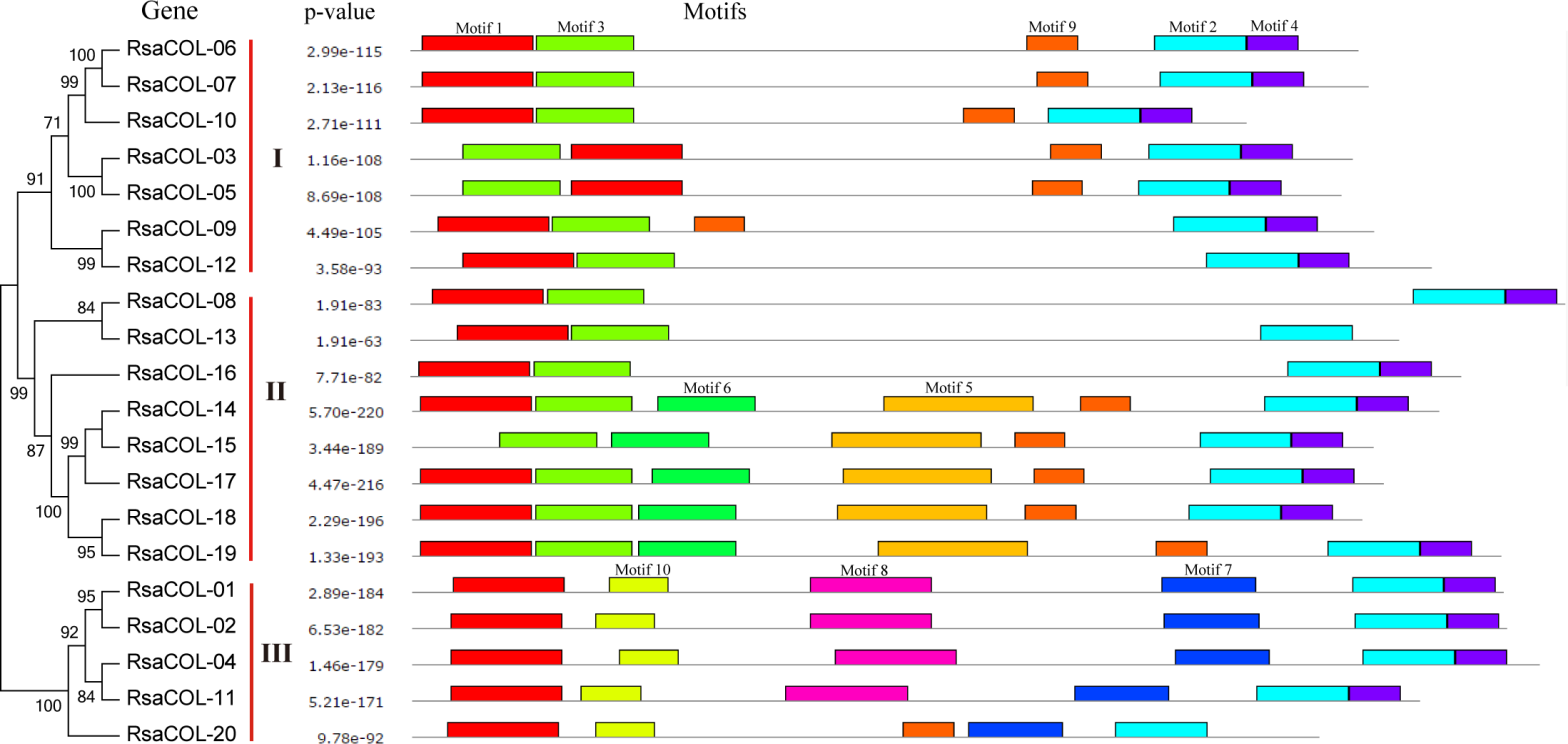
Conserved motifs embedded in the radish COL proteins. The phylogenetic tree, the number of conserved motifs and their distribution in each protein with their relative combined P-values.

**Fig 4:**
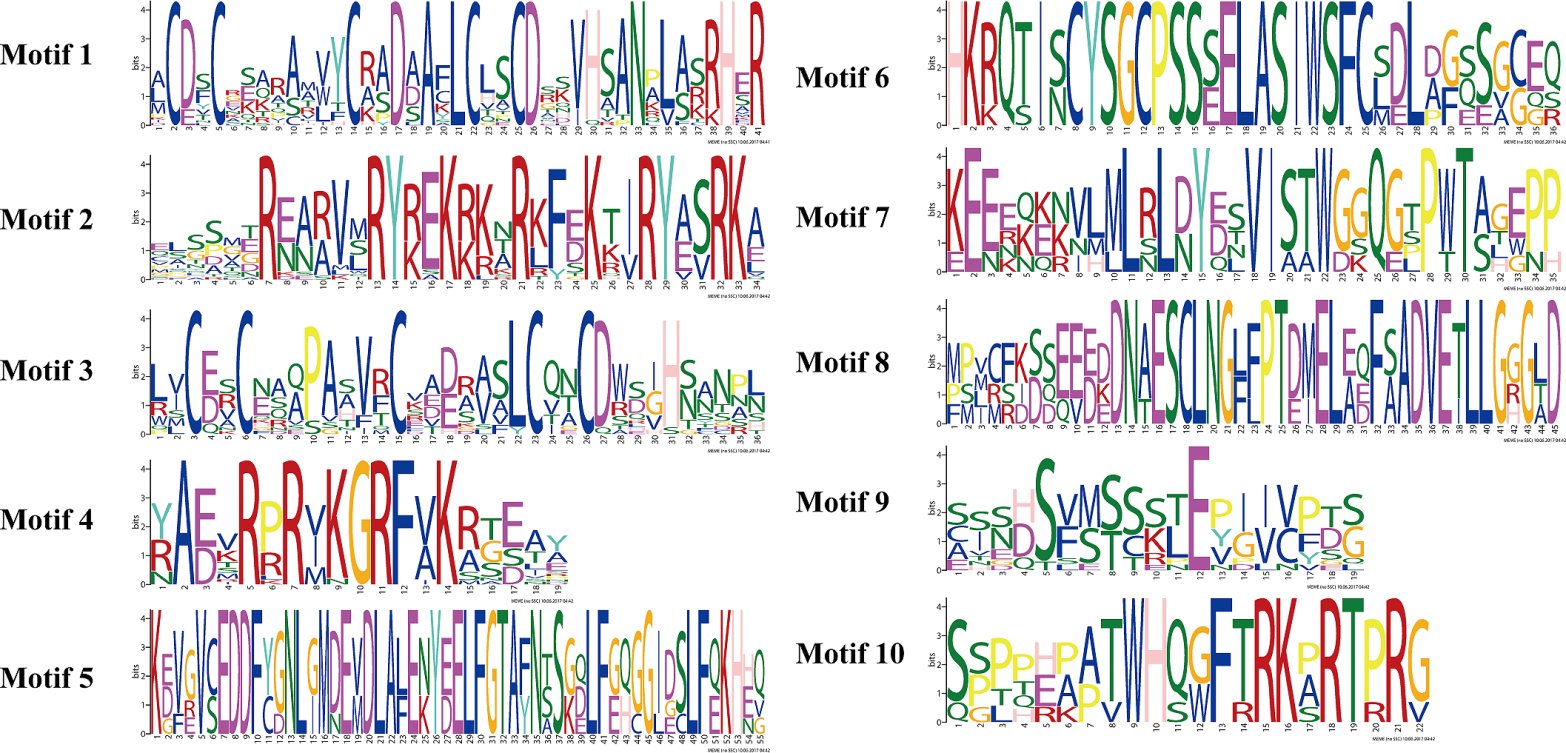
Amino acid sequences of each conserved motif in the radish COL proteins. The font size represents the frequency of the respective amino acid.

To explore the conserved domains and motifs, MEME software was employed to analyze the sequence alignment of the COL proteins in radish. The motifs were listed using serial numbers for Motif 1 to Motif 15 according to the ascending E-value of the alignment (Fig 3 and Fig 4). Motif 1 and Motif 2 were the most conserved motifs that could be found in all the COL proteins, followed by Motif 3 and Motif 4, which were conserved in 18 and 15 radish COL proteins, respectively, possibly because Motif 1 and Motif 2 were zinc finger domains and because Motif 3 was a CCT domain. Motif 3 is usually located nearly after Motif 1 in the protein sequences in Group I and Group II, except that Motif 1 was lost in RsaCOL-15 and the inversion events in RsaCOL-03 and RsaCOL-05. Motif 4 and Motif 9 were located in partial members of the *COL* genes in these three groups. Moreover, some motifs were only present in one unique group and were shared by all the members within the group. This was the case for Motif 7, Motif 8 and Motif 10 in Group III. In addition, Motif 6 was only found in 5 COL proteins in Group II; thus, it was the representative motif of Group II. For these motifs in radish COL proteins, the longest motif was 45 amino acids (aa), whereas the shortest motif was 19 aa.

### The orthologous, co-orthologous and paralogous *COL* genes in radish, *Arabidopsis*, rice and *P*. *patens*

To shed light on the vertical descent from a single ancestral gene and on duplication, a comparative analysis was performed to identify the orthologous, co-orthologous and paralogous gene pairs in radish, *Arabidopsis*, *Brassica rapa*, *Brassica oleracea*, *Capsella rubella* and rice. Orthologs are genes derived from a single gene (vertical descent) [51]. The meaning of co-orthologs is slightly different, in that they consist of two or more genes in one lineage that are collectively orthologous to one or more genes in another lineage due to lineage-specific duplication(s). Paralogous genes result from a lineage-specific duplication(s) that occurs subsequent to a given speciation event, and they are defined only relative to a speciation event, with no absolute meaning [52].

We identified 273 orthologous, 103 co-orthologous and 37 paralogous *COL* gene pairs among radish, *Arabidopsis*, *Brassica rapa*, *Brassica oleracea*, *Capsella rubella* and rice according to OrthoMCL software (S4 Table). In the network of homologous gene pairs, all the *COL* genes of radish, *Arabidopsis*, *Brassica oleracea* and *Capsella rubella* were included. A majority of *COL* genes in *Brassica rapa* and rice were included, except for two *Brassica rapa COL* genes and three rice *COL* genes. This homologous gene pair network was highly consistent with the phylogenetic tree, which could be clearly divided into three parts.

**Fig 5:**
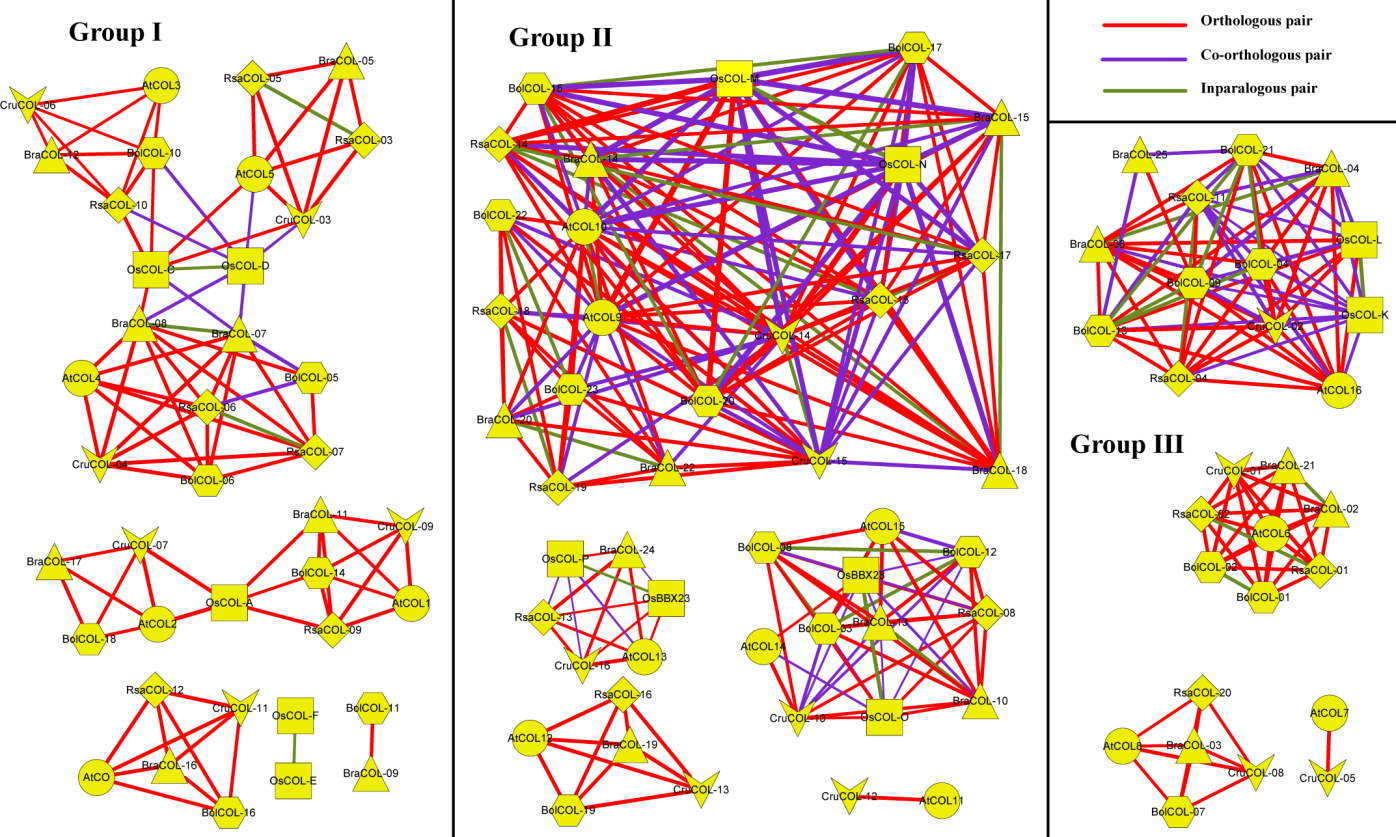
Orthologous, co-orthologous and paralogous *COL* gene pairs. The ellipse, diamond and rectangle shapes indicate genes belonging to radish, *Arabidopsis*, *Brassica rapa*, *Brassica oleracea*, *Capsella rubella* and rice. The red, purple and green lines indicate orthologous, co-orthologous and paralogous relationships, and the width of the line is associated with the relationship index as produced by OrthoMCL software.

For orthologous gene pairs, 19, 34, 33, 19, and 6 orthologous gene pairs were identified in groups of radish-*Arabidopsis*, radish-*Brassica rapa*, radish-*Brassica oleracea*, radish-*Capsella rubella* and radish-rice *COL* genes (Fig 5). In addition, 6, 1, 4, 6 and 11 co-orthologous gene pairs were identified in those groups, providing comparative information on *COL* genes among lower plants, eudicots and monocots. Generally, the ratio of orthologous pairs of closer plants with the radish in the phylogenetic tree exceeded the ratio of co-orthologous pairs. The *RsaCOL-03* and *RsaCOL-05* were two orthologs to *AtCOL5*, and their divergence time was 11.95 million years ago, illustrating that they were duplicated from a common ancestor with *AtCOL5* and that they diverged recently. Eight paralogous pairs were found in radish, with more than one pair in *Arabidopsis* and six in rice. There were no orthologous/co-orthologous genes for *AtCOL7* and *AtCOL11* in the *Brassica* genus and radish, whereas there was one counterpart for each gene in *Capsella rubella*, which indicated that there was *COL* gene loss in the *Brassica*-*Raphanus* lineage. This approach provides a better understanding of the general trends in the evolution of radish *COL* genes and of the reconstruction of the evolutionary history of each gene in its entirety.

### Selection and divergence time

Nonsynonymous (amino acid-replacing, Ka) and synonymous (Ks) substitution rates among protein-coding sequences were used to reveal the DNA sequence evolution mechanisms. The Ka/Ks ratio was used to estimate the selective strength for DNA sequence evolution, with a Ka/Ks > 1 indicating positive selection, Ka/Ks < 1 indicating purifying (negative) selection, and a Ka/Ks close to 1 indicating a neutral mutation [53]. ParaAT software (http://cbb.big.ac.cn/software) is capable of constructing multiple protein-coding DNA alignments in parallel for a large number of homologs [54]. To investigate the selection mechanisms of *COL* genes during evolution, we calculated the Ka, Ks and divergence time among the paralogous gene pairs obtained here (S4 Table). Notably, the homologous gene pairs were identified by OrthoMCL software using the ML method, whereas the Ka, Ks and divergence time were calculated based on the Nei and Gojobori method. This universally used method for alignment and Ks calculation was stricter in identifying the homologous gene pairs; therefore, some homologous gene pairs identified in OrthoMCL software were not regarded as homologous pairs by this method, and neither their Ks nor their divergence time were available. For example, the AtCOL16 and OsCOL-L were considered as orthologs by OrthoMCL software, whereas the Ks was not available by ParaAT and KaKs_Calculator software.

In Brassicaceae, three rounds of whole-genome duplication (WGD) occurred after its lineage diverged from the monocot lineage. The most recent WGD event occurred 50 to 65 MYA [55,56], prior to the divergence of the species in the Brassicaceae family. Several reports have estimated that the *Arabidopsis*-*Brassica* split took place 33 to 43 MYA. Notably, a further hexaploidization event (α’ whole-genome triplication [WGT] event occurred recently in the common ancestor of *Brassica* and *Raphanus*) occurred 22 to 29 MYA [55,57,58]. Subsequently, the α’ duplicates of the *Brassica* and *Raphanus* species shared ancestry over the following 5 to 16 million years ago (MYA), followed by 13 to 19 MYA of independent evolution in the *Brassica* and *Raphanus* genera [59]. Studies on the *Brassica* species suggested that after α’ WGT, >50% of the *Brassica* duplicate genes may have been lost via deletion and pseudogenization; part of them might have been lost in a biased fashion [60], and *Raphanus* species might have evolved along a similar path. There were 17 *COL* genes in *Arabidopsis*, which were assumed to be 54 duplicated genes immediately after a WGT event. However, only 37% (20 genes) were retained. The case of the *COL* genes would provide a better understanding of duplicate evolution post-WGT regarding the rate of pseudogenization in duplicate genes and the patterns of expression divergence in *Raphanus*.

Almost all calculated homologous *COL* gene pairs in the selected six plants had a Ka/Ks ratio of less than 1, indicating the purifying selection of these genes. The exception was the BolCOL-09 and BraCOL-25 gene pair, with a Ka/Ks ratio of 1.05, suggesting that the gene pair might go through neutral mutation. The Ka/Ks ratio of the only existing paralogous gene pair (*AtCOL9*-*AtCOL10*) in *Arabidopsis* was 0.19, and the ratio of the radish paralogous gene pair fell into a range from 0.12 to 0.32. The divergence times of the paralogous gene pairs in *Arabidopsis* and *Capsella rubella* were 31.03 and 35.99 MYA, respectively. The divergence times of the radish *COL* paralogous gene pairs ranged from 11.95 to 19.54 MYA, which occurred during a WGT event. The divergence time of the paralogous gene pairs in *Brassica rapa* was 9.05 to 18.50 MYA, while the divergence time of paralogous gene pairs in *Brassica oleracea* was 1.92 to 16.62 MYA.

The divergence times of a large portion of the orthologous gene pairs between radish-*Arabidopsis*, radish-*Capsella rubella* and radish-*Brassica* species were 10.97 to 28.39 MYA, 15.13 to 32.51 MYA, and 3.63 to 19.98 MYA, respectively. These findings suggested those orthologs with *Arabidopsis* and *Capsella rubella* duplications occurred during an α’ WGT event (22–29 MYA), the shared ancestry era of *Brassica* and *Raphanus* as well as an independent evolution era (13–19 MYA), whereas those orthologs with *Brassica* species occurred during an independent evolution era.

### Predicted regulating network of radish COL proteins

**Fig 6:**
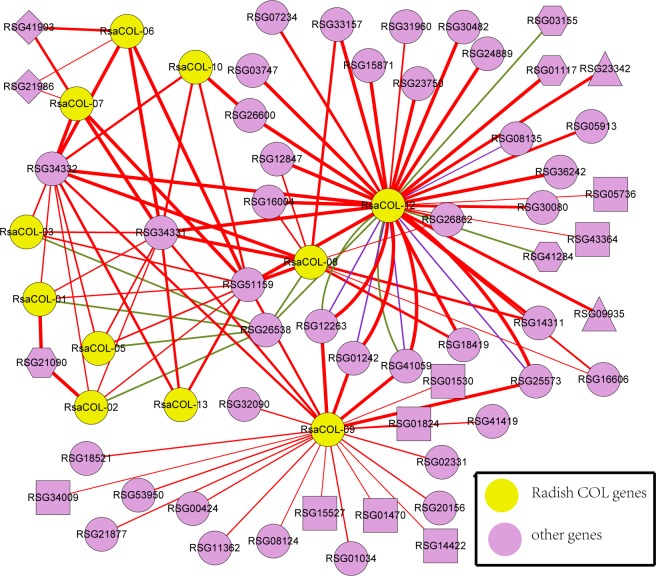
The interaction network of *COL* genes in radish according to the orthologs in *Arabidopsis*. Red and blue indicate that the Pearson correlation coefficient (PCC) index is above or below 0, respectively; green indicates that the PCC index of the interaction was unclear. The ellipse, diamond, triangle, rectangle and hexagon shapes indicate that this protein might be detected in the nucleus, vacuole, plastid, cytosol or unclear, respectively. If a protein could be found in several cell parts, including the nucleus, it is only represented by an ellipse.

To better understand the regulating network in which radish *COL* genes are involved, an interaction network of radish *COL* genes was constructed using a computational approach. The Pearson correlation coefficient values of over one hundred gene pairs were larger than zero, whereas the PCC of four genes were less than zero, which indicates that COL proteins primarily have a positive interaction with other proteins in radish. Ten gene pairs were not calculated, and they are thus shown in green lines, indicating that their regulation patterns were unclear. The results showed that the radish *COL* genes played significantly different roles in the interaction-regulating network (Fig 6). For example, RsaCOL-12 and RsaCOL-09 interacted with 32 and 23 proteins, respectively, suggesting that the two genes have significant regulation effects at the transcriptional level. The *RSG26538* gene was homologous to the *AtBBX32* in *Arabidopsis*, which was a B-box domain transcription factor. It was predicted to be involved in the blue light signaling pathway, the negative regulation of transcription, the DNA-templated photomorphogenesis, the red or far-red light signaling pathway, the regulation of flower development, the regulation of transcription, the DNA-templated response to chitin, transcription, and being DNA-templated by GO enrichment analysis. In *Arabidopsis*, the *AtCOL3* could target *FT* in the presence of *AtBBX32* to regulate the flowering pathway, thus playing a role at the interface between the light and the clock to modulate the timing and duration of specific reproductive stages [61]. Therefore, we hypothesized that *RsaCOL-03-RSG26538* was a corresponding module of *BBX32-COL3*, and it might participate in the regulatory mechanism affecting reproductive development in radish. Additionally, the transgenic of the *Arabidopsis AtBBX32* and overexpression in soybean significantly increased the grain yield in soybeans, which were potentially caused by the timing of reproductive development in transgenic soybeans, leading to the increased duration of the pod and seed development period. At the molecular level, *AtBBX32* influenced the transcript levels of the soybean clock genes *GmTOC1* and *LHY-CCA1-like2* (*GmLCL2*) and the clock-regulating gene *CO* [62]. Because these clock genes played vital roles in regulating many key agronomic traits, it would be worthwhile to use this approach as an efficient strategy for genetic manipulation in this regulatory pathway for increased radish agricultural productivity.

Both *RSG34331* and *RSG51159* interacted with 11 radish *COL* proteins, including *RsaCOL-01* to *RsaCOL-03*, *RsaCOL-05* to *RsaCOL-10*, *RsaCOL-12*, and *RsaCOL-13*. The annotation showed that *RSG3433*1 and *RSG51159* were both LOV kelch protein 2 (LKP2). Previous reports have demonstrated that the overexpression of LKP2 causes arrhythmia under both constant light and constant darkness, hypocotyl elongation, and postponed flowering under long days [63]. Therefore, we predicted that the radish LKP2s (*RSG34331* and *RSG51159*) were potentially close to the circadian oscillator, and there was a positive interaction among radish homologs and *AtCO*, *AtCO1*, *AtCO3*, *AtCO4*, *AtCO5*, *AtCO6*, *AtCO15*. The *RSG26538* had a positive interaction with *RsaCOL-09* and *RSG51159*, and an unclear interaction with *RsaCOL-01*, *RsaCOL-02*, *RsaCOL-03*, *RsaCOL-05*, *RsaCOL-08* and *RsaCOL-12*.

### Expression pattern of the radish *COL* genes during development

**Fig 7:**
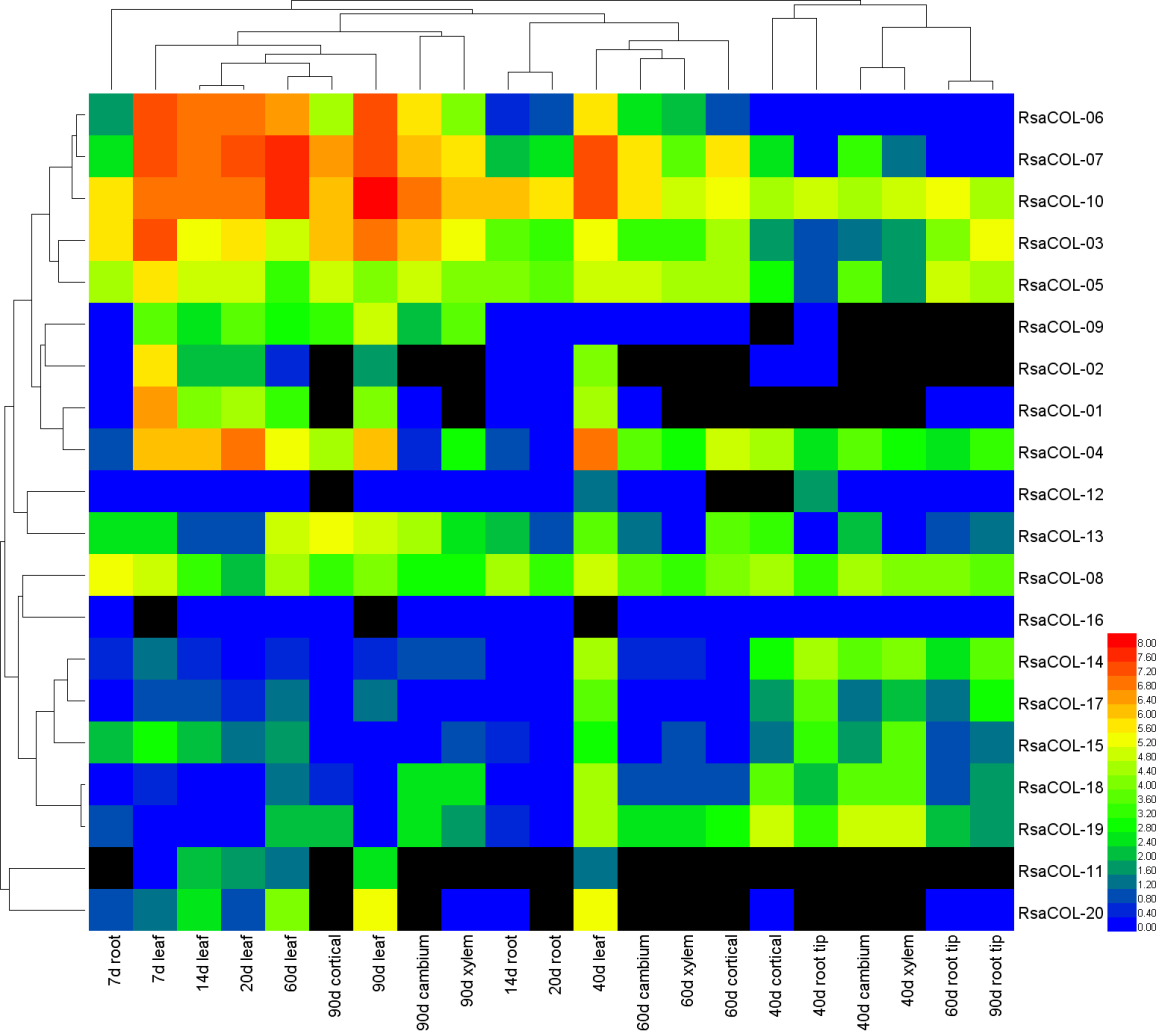
The heat map shows the expression profile of the radish *COL* genes over multiple development periods.

We investigated the expression of each *COL* gene using published RNA-seq data for different radish tissues during vegetative and reproductive development. The expression level of each gene was calculated using the RPKM method. We also analyzed the average and median expression values of all the genes in a full radish gene dataset that contained 64,657 Augustus gene models for comparison, which was stable in each sample and available for comparison across samples. The average expression value of all the radish genes was approximately 15, whereas the median expression value of all the radish coding genes ranged from 0.1 to 0.2 (S5 Table). According to the RPKM values, all the *RsaCOL* genes were expressed in the leaf, and 19 of these genes were expressed at relatively high levels (RPKM>1) in at least one tissue (Fig 7). The expression values of *RsaCOL-16* were lower in all the samples (lower than 0.34), suggesting that it may not act as a functional gene.

Clustering was performed using both horizontal and vertical axes. Interestingly, the clustering of the vertical axis including 20 radish *COL* genes was consistent with the phylogenetic tree. The genes *RsaCOL-03*, *RsaCOL-05*, *RsaCOL-06*, *RsaCOL-07*, *RsaCOL-09*, and *RsaCOL-10* in Group I were clustered closely in both the expression clustering and the phylogenetic tree; *RsaCOL-12* was also clustered relatively close to those genes. *RsaCOL-01*, *RsaCOL-02* and *RsaCOL-04* in Group III also illustrated a similar expression pattern, while *RsaCOL-11* and *RsaCOL-20* were clustered closely. The members in Group II were also significantly expressed following a similar pattern except for *RsaCOL-13*.

In general, the expression of radish *COL* genes in Group I and Group III decreased during development, whereas the expression of Group II *COL* genes in radish first increased and then decreased during development. In the leaves, the expression of *RsaCOL-06* was highest at 7 d (191.17), gradually decreased at 14 d (120.91) and 20 d (140.57), and reached the lowest at 40 d (61.98), then finally increased at 60 d and 90 d. Moreover, at the same developmental stage, the expression of *COL* genes in the leaf was higher than it was in the cortex, cambium and xylem, and thus higher than it was in the root. This finding could be a result of circadian clock receptor and coordination regulation. The identification and characterization of *RsaCOL* genes that regulate the circadian clock and flower development at the molecular level could contribute to an understanding of the genetic mechanisms underlying bolting and flowering in root vegetables and facilitate radish cultivar improvement.

The Pearson correlation coefficient of gene expression showed that nine COL radish proteins were highly co-expressed with 898 other radish proteins. RsaCOL-07 and RsaCOL-06 were observed to be co-expressed with 298 and 288 proteins, respectively. The RSG09940 was annotated as a set domain-containing protein, which was co-expressed with RsaCOL-06, RsaCOL-07, RsaCOL-10 and RsaCOL-11. RSG02061, RSG05030, RSG08140, RSG10915, RSG11552, RSG13476, RSG15344, RSG16356, RSG20934, RSG29339, RSG33164, RSG45245, and RSG51054 were co-expressed with 3 radish COL genes (S6 Table).

**Fig 8:**
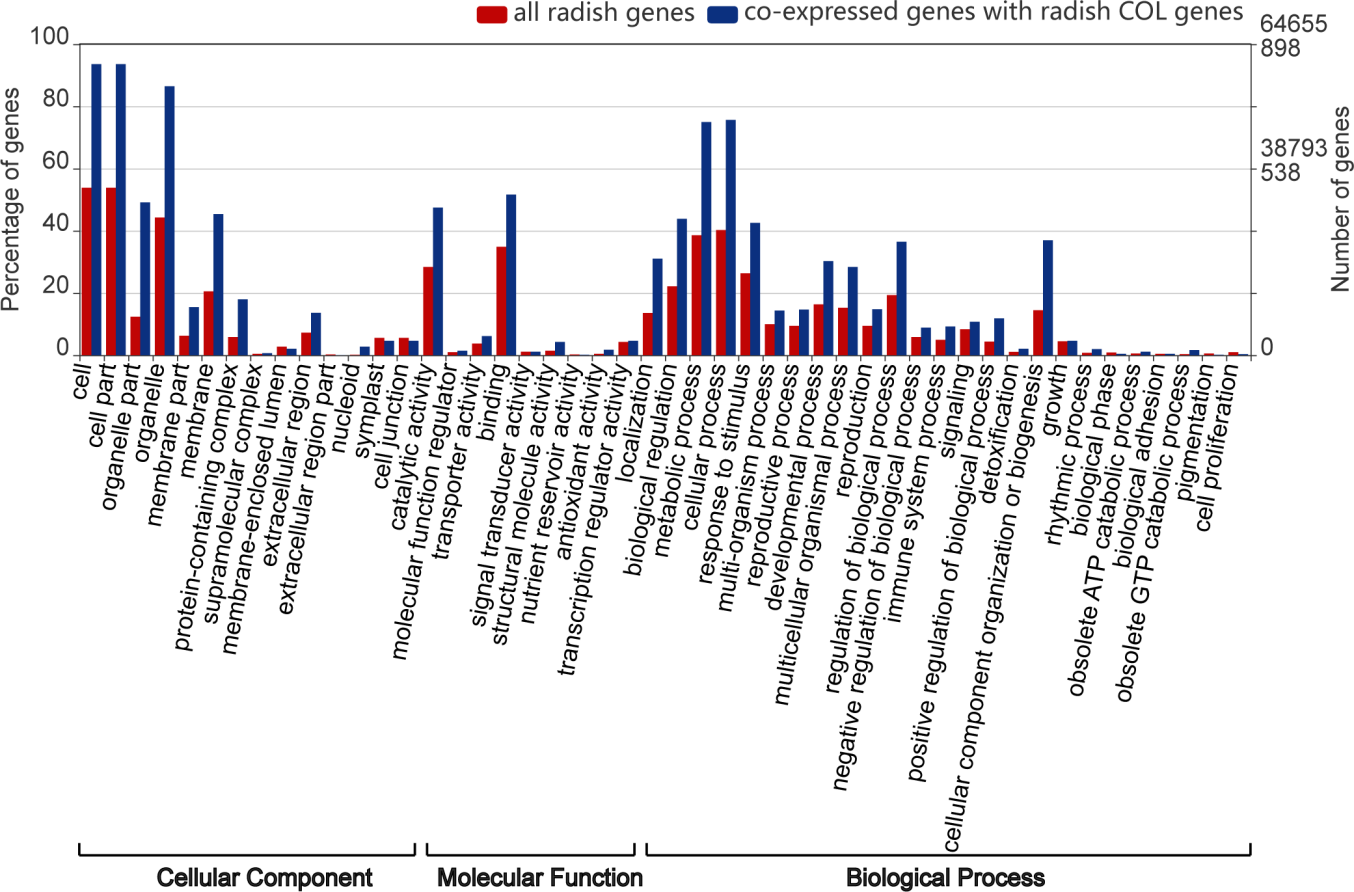
The GO annotation for all the radish coding genes and *COL* co-expressed genes.

To provide insight into the functions of newly identified radish genes, all radish genes and COL co-expressed genes were annotated using GO databases. A Gene Ontology (GO) enrichment analysis was performed to provide a dynamic, controlled vocabulary and hierarchical relationships for the information on molecular functions, cellular components and biological processes [64,65]. This approach provided important access to the underlying fundamental functions of new genes. In total, 42,347 out of 64,655 (65.50%) radish-coding genes were assigned with GO terms, whereas 867 out of 898 (96.55%) co-expressed genes were annotated (Fig 8, S7 Table). This result indicated that a large portion of those co-expressed COL genes were well-studied, and the ratio of co-expressed genes was higher than that of all the radish coding genes in substantial GO level 2 terms. The three most common categories for biological processes were cellular processes (679), metabolic processes (673) and biological regulation (393). For molecular functions, binding (463) and catalytic activity (426) were the two most abundant categories. For cellular components, the annotated genes involved in cell parts (840), cells (840) and organelles (782) were the most abundant entries.

### Expression pattern of the radish *COL* genes during development

**Fig 9:**
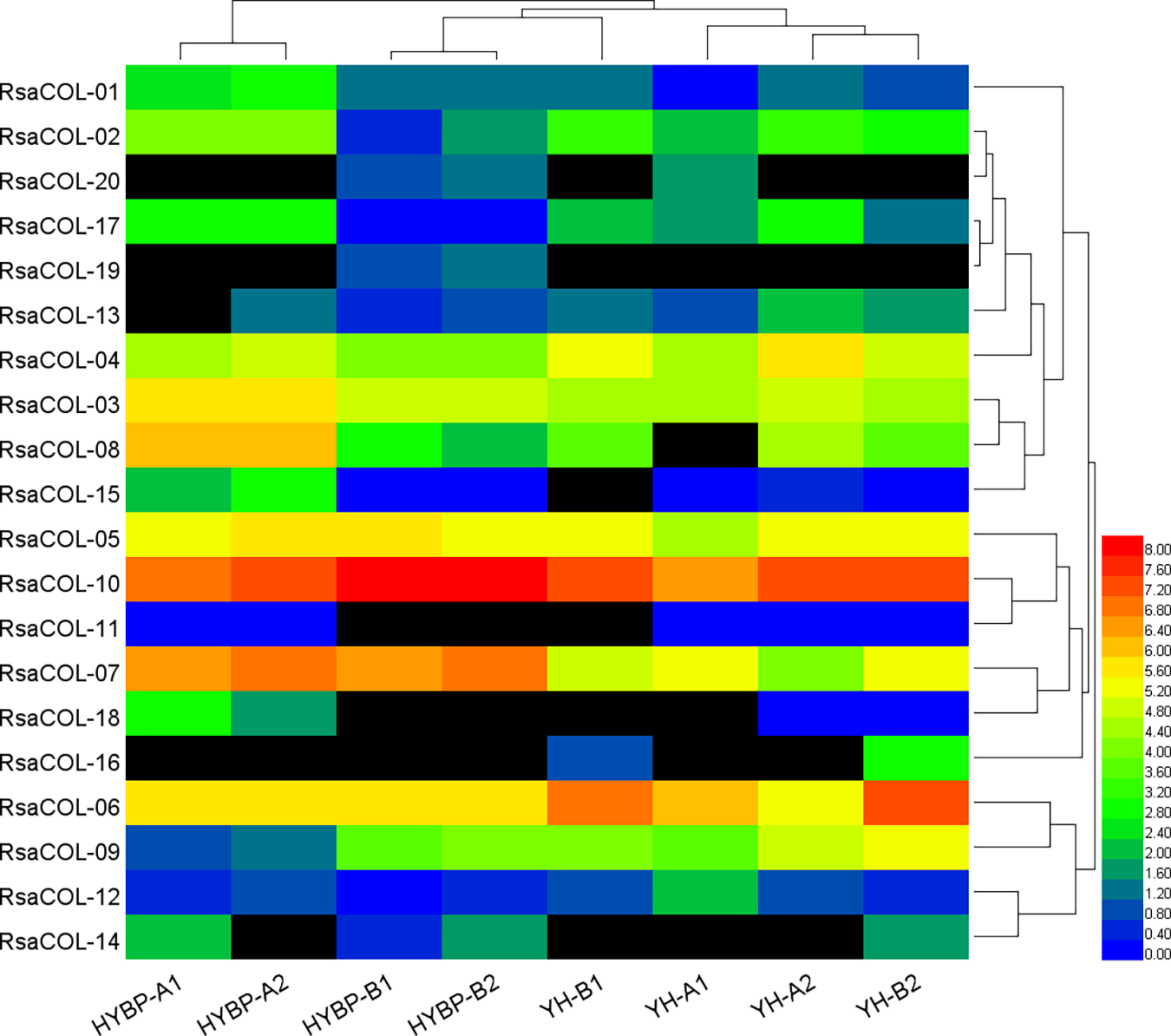
The heat map shows the expression profile of the *COL* genes in radish cytoplasmic male sterility and fertile floral buds.

We re-analyzed the expression of each *COL* gene in radish buds with lengths approximately 1.5 mm from two CMS lines (HYBP-B, YH-B) possessing the CMS-inducing orf138 gene and corresponding to near-isogenic maintainer lines (HYBP-A, YH-A) [43].The clustering of samples showed that the expression of COL genes was not associated with cytoplasmic male sterility (Fig 9, S8 Table). The expression of *RsaCOL-01*, *RsaCOL-02*, *RsaCOL-08*, *RsaCOL-15*, and *RsaCOL-17* significantly declined in CMS line HYBP-B compared with normal line HYBP-A. However, the expression of those genes was insignificantly expressed in CMS line YH-B. The expression of *RsaCOL-09* increased in HYBP-B, and the expression of *RsaCOL-06* increased in YH-B. These genes were non-significantly expressed in another CMS line, which indicated that the expression levels of the *COL* genes were not coincident in those two CMS lines. The expression levels of radish *COL* genes were more closely associated with the cultivars, more than with whether the plant was from a CMS line or not.

### Expression pattern of the radish *COL* genes in response to vernalization

**Fig 10:**
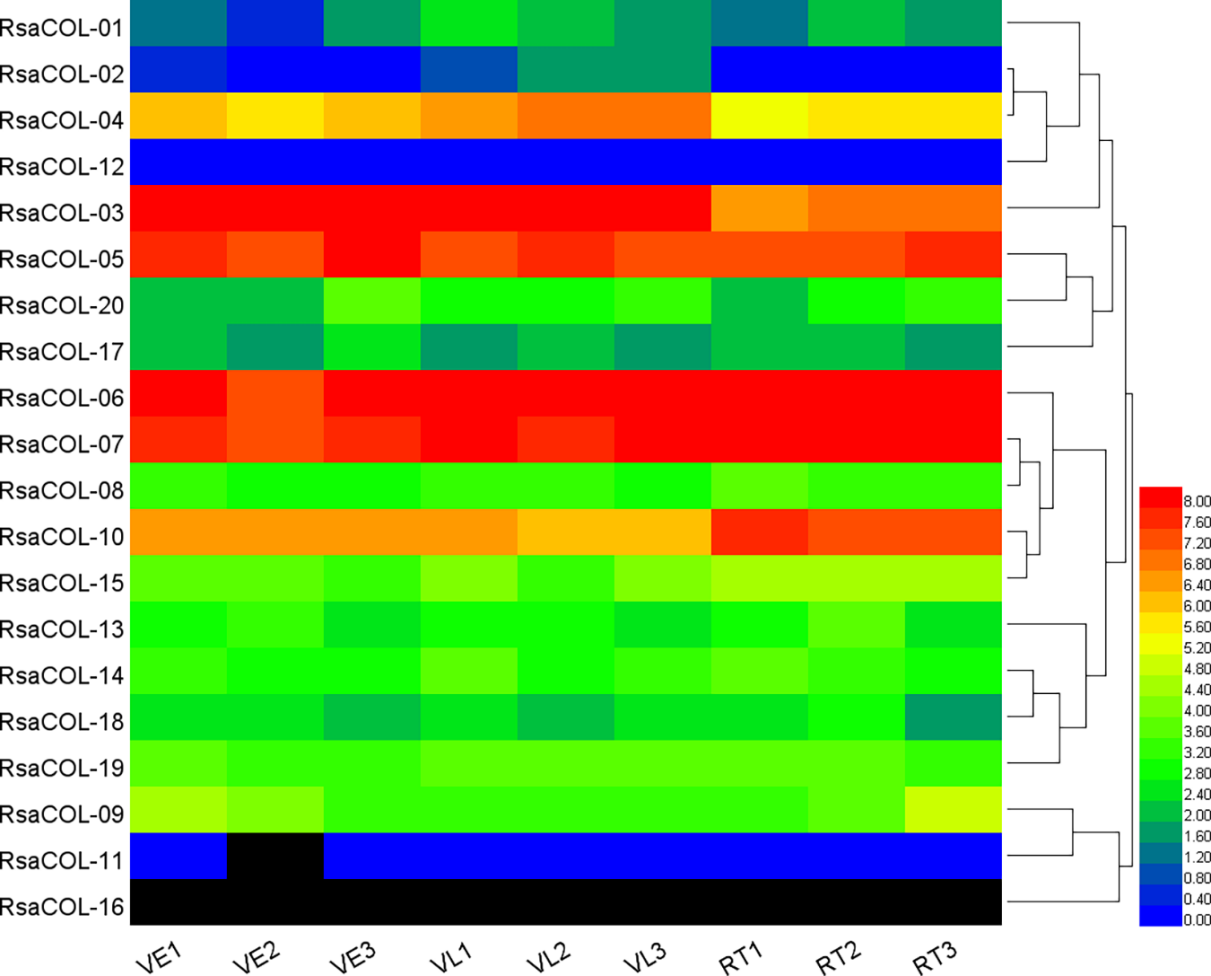
The heat map shows the expression profile of the radish *COL* genes of radish in response to vernalization.

To investigate the potential links between *COL* genes and radish vernalization, we determined the expression of each *COL* gene from radish plants that were exposed to low temperatures for 0, 3 and 20 d in a previous report [44]. Substantial numbers of radish *COL* genes were differentially expressed after vernalization treatment (Fig 10, S9 Table). The expression of *RsaCOL-03*, *RsaCOL-04*, *RsaCOL-05*, *RsaCOL-06*, *RsaCOL-07* and *RsaCOL-10* were high in all the samples, of which most were from Group I. The expression levels of *RsaCOL-02* and *RsaCOL-04* were significantly increased during vernalization treatment, while the expression of *RsaCOL-10* was significantly decreased. Using *COL5* over-expressing lines, we show that, under short days, the constitutive expression of *COL5* affects the flowering time and the expression of the floral integrator genes *FT* and *SOC1* [66]. The expression of the corresponding radish gene *RsaCOL-03* was significantly increased at the early and late stages of vernalization. *RsaCOL-14* and *RsaCOL-15* were orthologs corresponding to Arabidopsis gene *COL9*, which is regulated by the circadian clock in the photoperiod pathway, by repressing the expression of *CO* and concomitantly reducing the expression of *FT*, delaying floral transition [67]. The expression of *RsaCOL-15* halved at the early stage of vernalization and then remained steady at the late stage of vernalization, while *RsaCOL-14* was not differentially expressed. The *RsaCOL-05*, an ortholog of *COL5* in *Arabidopsis*, remained steady during all the stages of the vernalization treatment.

## Supporting information

S1 Fig Map of *COL* genes in radish chromosomes. (PDF)

S1 Table Location and sequences of radish *COL* genes. (XLSX)

S2 Table Location and sequences of COL genes in the radish cultivar ‘kazusa’. (XLSX)

S3 Table *COL* genes identified in representative plants. (XLSX)

S4 Table Homologous gene pairs and their Ka, Ks and divergence time. (XLSX)

S5 Table Expression of radish *COL* genes in tissues throughout the development stages. (XLSX)

S6 Table PCC values showed the co-expression of *COL* genes and other genes in radish. (XLSX)

S7 Table GO annotation for the radish *COL* co-expressed genes. (XLSX)

S8 Table Expression of *COL* genes of radish during cytoplasmic male sterility. (XLSX)

S9 Table Expression of *COL* genes of radish during the response to vernalization. (XLSX)

## References

[1] Amasino RM, Michaels SD (2010) The timing of flowering. Plant Physiol 154: 516–520. 10.1104/pp.110.161653 20921176 PMC2948982

[2] Nie S, Xu L, Wang Y, Huang D, Muleke EM, Sun X, et al (2015) Identification of bolting-related microRNAs and their targets reveals complex miRNA-mediated flowering-time regulatory networks in radish (Raphanus sativus L.). Sci Rep 5: 14034 10.1038/srep14034 26369897 PMC4570191

[3] Srikanth A, Schmid M (2011) Regulation of flowering time: all roads lead to Rome. Cell Mol Life Sci 68: 2013–2037. 10.1007/s00018-011-0673-y 21611891 PMC11115107

[4] Fornara F, de Montaigu A, Coupland G (2010) SnapShot: Control of flowering in Arabidopsis. Cell 141: 550, 550 e551–552. 10.1016/j.cell.2010.04.024 20434991

[5] Michaels SD, Amasino RM (1999) FLOWERING LOCUS C encodes a novel MADS domain protein that acts as a repressor of flowering. Plant Cell 11: 949–956. 10330478 10.1105/tpc.11.5.949 PMC144226

[6] Lee J, Lee I (2010) Regulation and function of SOC1, a flowering pathway integrator. J Exp Bot 61: 2247–2254. 10.1093/jxb/erq098 20413527

[7] Moon J, Lee H, Kim M, Lee I (2005) Analysis of flowering pathway integrators in Arabidopsis. Plant Cell Physiol 46: 292–299. 10.1093/pcp/pci024 15695467

[8] Parcy F (2005) Flowering: a time for integration. Int J Dev Biol 49: 585–593. 10.1387/ijdb.041930fp 16096967

[9] Putterill J, Robson F, Lee K, Simon R, Coupland G (1995) The CONSTANS gene of Arabidopsis promotes flowering and encodes a protein showing similarities to zinc finger transcription factors. Cell 80: 847–857. 7697715 10.1016/0092-8674(95)90288-0

[10] Samach A, Onouchi H, Gold SE, Ditta GS, Schwarz-Sommer Z, Yanofsky MF, et al (2000) Distinct roles of CONSTANS target genes in reproductive development of Arabidopsis. Science 288: 1613–1616. 10834834 10.1126/science.288.5471.1613

[11] Suarez-Lopez P, Wheatley K, Robson F, Onouchi H, Valverde F, Coupland G (2001) CONSTANS mediates between the circadian clock and the control of flowering in Arabidopsis. Nature 410: 1116–1120. 10.1038/35074138 11323677

[12] An H, Roussot C, Suarez-Lopez P, Corbesier L, Vincent C, Pineiro M, et al (2004) CONSTANS acts in the phloem to regulate a systemic signal that induces photoperiodic flowering of Arabidopsis. Development 131: 3615–3626. 10.1242/dev.01231 15229176

[13] Robson F, Costa MM, Hepworth SR, Vizir I, Pineiro M, Reeves PH, et al (2001) Functional importance of conserved domains in the flowering-time gene CONSTANS demonstrated by analysis of mutant alleles and transgenic plants. Plant J 28: 619–631. 11851908 10.1046/j.1365-313x.2001.01163.x

[14] Borden KL (2000) RING domains: master builders of molecular scaffolds? J Mol Biol 295: 1103–1112. 10.1006/jmbi.1999.3429 10653689

[15] Khanna R, Kronmiller B, Maszle DR, Coupland G, Holm M, Mizuno T, et al (2009) The Arabidopsis B-box zinc finger family. Plant Cell 21: 3416–3420. 10.1105/tpc.109.069088 19920209 PMC2798317

[16] Cockram J, Thiel T, Steuernagel B, Stein N, Taudien S, Bailey PC, et al (2012) Genome dynamics explain the evolution of flowering time CCT domain gene families in the Poaceae. PLoS One 7: e45307 10.1371/journal.pone.0045307 23028921 PMC3454399

[17] Wu W, Zheng XM, Chen D, Zhang Y, Ma W, Zhang H, et al (2017) OsCOL16, encoding a CONSTANS-like protein, represses flowering by up-regulating Ghd7 expression in rice. Plant Sci 260: 60–69. 10.1016/j.plantsci.2017.04.004 28554475

[18] Jin JP, Zhang H, Kong L, Gao G, Luo JC (2014) PlantTFDB 3.0: a portal for the functional and evolutionary study of plant transcription factors. Nucleic Acids Research 42: D1182–D1187. 10.1093/nar/gkt1016 24174544 PMC3965000

[19] Griffiths S, Dunford RP, Coupland G, Laurie DA (2003) The evolution of CONSTANS-like gene families in barley, rice, and Arabidopsis. Plant Physiol 131: 1855–1867. 10.1104/pp.102.016188 12692345 PMC166942

[20] Song X, Duan W, Huang Z, Liu G, Wu P, Liu T, et al (2015) Comprehensive analysis of the flowering genes in Chinese cabbage and examination of evolutionary pattern of CO-like genes in plant kingdom. Sci Rep 5: 14631 10.1038/srep14631 26416765 PMC4586889

[21] Curtis IS (2003) The noble radish: past, present and future. Trends Plant Sci 8: 305–307. 10.1016/S1360-1385(03)00127-4 12878009

[22] Mitsui Y, Shimomura M, Komatsu K, Namiki N, Shibata-Hatta M, Imai M, et al (2015) The radish genome and comprehensive gene expression profile of tuberous root formation and development. Scientific Reports 5. 10.1038/srep10835 PMC4650646 26056784

[23] Jeong YM, Kim N, Ahn BO, Oh M, Chung WH, Chung H, et al (2016) Elucidating the triplicated ancestral genome structure of radish based on chromosome-level comparison with the Brassica genomes. Theor Appl Genet 129: 1357–1372. 10.1007/s00122-016-2708-0 27038817

[24] Wang SF, Wang XF, He QW, Liu XX, Xu WL, Li LB, et al (2012) Transcriptome analysis of the roots at early and late seedling stages using Illumina paired-end sequencing and development of EST-SSR markers in radish. Plant Cell Reports 31: 1437–1447. 10.1007/s00299-012-1259-3 22476438

[25] Wang Y, Pan Y, Liu Z, Zhu X, Zhai L, Xu L, et al (2013) De novo transcriptome sequencing of radish (Raphanus sativus L.) and analysis of major genes involved in glucosinolate metabolism. BMC Genomics 14: 836 10.1186/1471-2164-14-836 24279309 PMC4046679

[26] Xu L, Wang Y, Liu W, Wang J, Zhu X, Zhang K, et al (2015) De novo sequencing of root transcriptome reveals complex cadmium-responsive regulatory networks in radish (Raphanus sativus L.). Plant Sci 236: 313–323. 10.1016/j.plantsci.2015.04.015 26025544

[27] Zhang L, Jia H, Yin Y, Wu G, Xia H, Wang X, et al (2013) Transcriptome analysis of leaf tissue of Raphanus sativus by RNA sequencing. PLoS One 8: e80350 10.1371/journal.pone.0080350 24265813 PMC3827192

[28] Curtis IS, Nam HG, Yun JY, Seo KH (2002) Expression of an antisense GIGANTEA (GI) gene fragment in transgenic radish causes delayed bolting and flowering. Transgenic Res 11: 249–256. 12113457 10.1023/a:1015655606996

[29] Nie S, Li C, Wang Y, Xu L, Muleke EM, Tang M, et al (2016) Transcriptomic Analysis Identifies Differentially Expressed Genes (DEGs) Associated with Bolting and Flowering in Radish (Raphanus sativus L.). Front Plant Sci 7: 682 10.3389/fpls.2016.00682 27252709 PMC4877535

[30] Finn RD, Bateman A, Clements J, Coggill P, Eberhardt RY, Eddy SR, et al (2014) Pfam: the protein families database. Nucleic Acids Research 42: D222–D230. 10.1093/nar/gkt1223 24288371 PMC3965110

[31] Eddy SR (2011) Accelerated Profile HMM Searches. Plos Computational Biology 7. 10.1371/journal.pcbi.1002195 PMC3197634 22039361

[32] Goodstein DM, Shu S, Howson R, Neupane R, Hayes RD, Fazo J, et al (2012) Phytozome: a comparative platform for green plant genomics. Nucleic Acids Res 40: D1178–1186. 10.1093/nar/gkr944 22110026 PMC3245001

[33] Nystedt B, Street NR, Wetterbom A, Zuccolo A, Lin YC, Scofield DG, et al (2013) The Norway spruce genome sequence and conifer genome evolution. Nature 497: 579–584. 10.1038/nature12211 23698360

[34] Wang K, Deng J, Damaris RN, Yang M, Xu L, Yang P (2015) LOTUS-DB: an integrative and interactive database for Nelumbo nucifera study. Database (Oxford) 2015: bav023. 25819075 10.1093/database/bav023 PMC4383347

[35] Gasteiger E, Gattiker A, Hoogland C, Ivanyi I, Appel RD, Bairoch A (2003) ExPASy: The proteomics server for in-depth protein knowledge and analysis. Nucleic Acids Res 31: 3784–3788. 12824418 10.1093/nar/gkg563 PMC168970

[36] Larkin MA, Blackshields G, Brown NP, Chenna R, McGettigan PA, McWilliam H, et al (2007) Clustal W and Clustal X version 2.0. Bioinformatics 23: 2947–2948. 10.1093/bioinformatics/btm404 17846036

[37] Tamura K, Stecher G, Peterson D, Filipski A, Kumar S (2013) MEGA6: Molecular Evolutionary Genetics Analysis version 6.0. Mol Biol Evol 30: 2725–2729. 10.1093/molbev/mst197 24132122 PMC3840312

[38] Hu B, Jin J, Guo AY, Zhang H, Luo J, Gao G (2015) GSDS 2.0: an upgraded gene feature visualization server. Bioinformatics 31: 1296–1297. 10.1093/bioinformatics/btu817 25504850 PMC4393523

[39] Bailey TL, Boden M, Buske FA, Frith M, Grant CE, Clementi L, et al (2009) MEME SUITE: tools for motif discovery and searching. Nucleic Acids Res 37: W202–208. 10.1093/nar/gkp335 19458158 PMC2703892

[40] Li L, Stoeckert CJ Jr., Roos DS (2003) OrthoMCL: identification of ortholog groups for eukaryotic genomes. Genome Res 13: 2178–2189. 10.1101/gr.1224503 12952885 PMC403725

[41] Koch MA, Haubold B, Mitchell-Olds T (2000) Comparative evolutionary analysis of chalcone synthase and alcohol dehydrogenase loci in Arabidopsis, Arabis, and related genera (Brassicaceae). Mol Biol Evol 17: 1483–1498. 10.1093/oxfordjournals.molbev.a026248 11018155

[42] Smoot ME, Ono K, Ruscheinski J, Wang PL, Ideker T (2011) Cytoscape 2.8: new features for data integration and network visualization. Bioinformatics 27: 431–432. 10.1093/bioinformatics/btq675 21149340 PMC3031041

[43] Mei S, Liu T, Wang Z (2016) Comparative Transcriptome Profile of the Cytoplasmic Male Sterile and Fertile Floral Buds of Radish (Raphanus sativus L.). Int J Mol Sci 17. 10.3390/ijms17010042 PMC4730287 26751440

[44] Liu C, Wang S, Xu W, Liu X (2017) Genome-wide transcriptome profiling of radish (Raphanus sativus L.) in response to vernalization. PLoS One 12: e0177594 10.1371/journal.pone.0177594 28498850 PMC5428929

[45] Ye J, Fang L, Zheng H, Zhang Y, Chen J, Zhang Z, et al (2006) WEGO: a web tool for plotting GO annotations. Nucleic Acids Res 34: W293–297. 10.1093/nar/gkl031 16845012 PMC1538768

[46] Kitashiba H, Li F, Hirakawa H, Kawanabe T, Zou Z, Hasegawa Y, et al (2014) Draft sequences of the radish (Raphanus sativus L.) genome. DNA Res 21: 481–490. 10.1093/dnares/dsu014 24848699 PMC4195494

[47] Jeong YM, Chung WH, Chung H, Kim N, Park BS, Lim KB, et al (2014) Comparative analysis of the radish genome based on a conserved ortholog set (COS) of Brassica. Theor Appl Genet 127: 1975–1989. 10.1007/s00122-014-2354-3 25056003

[48] Karanja BK, Fan L, Xu L, Wang Y, Zhu X, Tang M, et al (2017) Genome-wide characterization of the WRKY gene family in radish (Raphanus sativus L.) reveals its critical functions under different abiotic stresses. Plant Cell Rep 36: 1757–1773. 10.1007/s00299-017-2190-4 28819820

[49] Li C, Wang Y, Xu L, Nie S, Chen Y, Liang D, et al (2016) Genome-Wide Characterization of the MADS-Box Gene Family in Radish (Raphanus sativus L.) and Assessment of Its Roles in Flowering and Floral Organogenesis. Front Plant Sci 7: 1390 10.3389/fpls.2016.01390 27703461 PMC5028395

[50] Zobell O, Coupland G, Reiss B (2005) The family of CONSTANS-like genes in Physcomitrella patens. Plant Biol (Stuttg) 7: 266–275. 15912446 10.1055/s-2005-865621

[51] Fitch WM (1970) Distinguishing homologous from analogous proteins. Syst Zool 19: 99–113. 5449325

[52] Koonin EV (2005) Orthologs, paralogs, and evolutionary genomics. Annu Rev Genet 39: 309–338. 10.1146/annurev.genet.39.073003.114725 16285863

[53] Zhang Z, Yu J (2006) Evaluation of six methods for estimating synonymous and nonsynonymous substitution rates. Genomics Proteomics Bioinformatics 4: 173–181. 10.1016/S1672-0229(06)60030-2 17127215 PMC5054070

[54] Zhang Z, Xiao J, Wu J, Zhang H, Liu G, Wang X, et al (2012) ParaAT: a parallel tool for constructing multiple protein-coding DNA alignments. Biochem Biophys Res Commun 419: 779–781. 10.1016/j.bbrc.2012.02.101 22390928

[55] Beilstein MA, Nagalingum NS, Clements MD, Manchester SR, Mathews S (2010) Dated molecular phylogenies indicate a Miocene origin for Arabidopsis thaliana. Proc Natl Acad Sci U S A 107: 18724–18728. 10.1073/pnas.0909766107 20921408 PMC2973009

[56] Bowers JE, Chapman BA, Rong J, Paterson AH (2003) Unravelling angiosperm genome evolution by phylogenetic analysis of chromosomal duplication events. Nature 422: 433–438. 10.1038/nature01521 12660784

[57] Town CD, Cheung F, Maiti R, Crabtree J, Haas BJ, Wortman JR, et al (2006) Comparative genomics of Brassica oleracea and Arabidopsis thaliana reveal gene loss, fragmentation, and dispersal after polyploidy. Plant Cell 18: 1348–1359. 10.1105/tpc.106.041665 16632643 PMC1475499

[58] Couvreur TL, Franzke A, Al-Shehbaz IA, Bakker FT, Koch MA, Mummenhoff K (2010) Molecular phylogenetics, temporal diversification, and principles of evolution in the mustard family (Brassicaceae). Mol Biol Evol 27: 55–71. 10.1093/molbev/msp202 19744998

[59] Moghe GD, Hufnagel DE, Tang H, Xiao Y, Dworkin I, Town CD, et al (2014) Consequences of Whole-Genome Triplication as Revealed by Comparative Genomic Analyses of the Wild Radish Raphanus raphanistrum and Three Other Brassicaceae Species. Plant Cell 26: 1925–1937. 10.1105/tpc.114.124297 24876251 PMC4079359

[60] Wang X, Wang H, Wang J, Sun R, Wu J, Liu S, et al (2011) The genome of the mesopolyploid crop species Brassica rapa. Nat Genet 43: 1035–1039. 10.1038/ng.919 21873998

[61] Tripathi P, Carvallo M, Hamilton EE, Preuss S, Kay SA (2017) Arabidopsis B-BOX32 interacts with CONSTANS-LIKE3 to regulate flowering. Proc Natl Acad Sci U S A 114: 172–177. 10.1073/pnas.1616459114 27999181 PMC5224386

[62] Preuss SB, Meister R, Xu Q, Urwin CP, Tripodi FA, Screen SE, et al (2012) Expression of the Arabidopsis thaliana BBX32 gene in soybean increases grain yield. PLoS One 7: e30717 10.1371/journal.pone.0030717 22363475 PMC3281879

[63] Schultz TF, Kiyosue T, Yanovsky M, Wada M, Kay SA (2001) A role for LKP2 in the circadian clock of Arabidopsis. Plant Cell 13: 2659–2670. 10.1105/tpc.010332 11752379 PMC139480

[64] Ashburner M, Ball CA, Blake JA, Botstein D, Butler H, Cherry JM, et al (2000) Gene ontology: tool for the unification of biology. The Gene Ontology Consortium. Nat Genet 25: 25–29. 10.1038/75556 10802651 PMC3037419

[65] Gene Ontology Consortium Blake JA, Dolan M, Drabkin H, Hill DP, Li N, et al (2013) Gene Ontology annotations and resources. Nucleic Acids Res 41: D530—D535. 10.1093/nar/gks1050 23161678 PMC3531070

[66] Hassidim M, Harir Y, Yakir E, Kron I, Green RM (2009) Over-expression of CONSTANS-LIKE 5 can induce flowering in short-day grown Arabidopsis. Planta 230: 481–491. 10.1007/s00425-009-0958-7 19504268

[67] Cheng XF, Wang ZY (2005) Overexpression of COL9, a CONSTANS-LIKE gene, delays flowering by reducing expression of CO and FT in Arabidopsis thaliana. Plant J 43: 758–768. 10.1111/j.1365-313X.2005.02491.x 16115071

