# Supplementary figures and images for: Genome-wide identification and characterization of CONSTANS-like gene family in radish (Raphanus sativus)
Source: PLoS One. 2018 Sep 24;13(9):e0204137. doi: 10.1371/journal.pone.0204137 (PMC6152963; doi:10.1371/journal.pone.0204137)

**R1**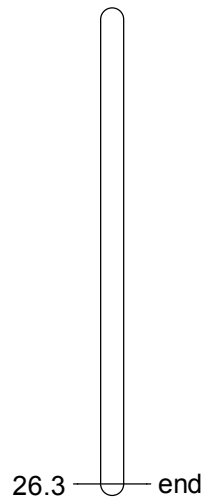**R2**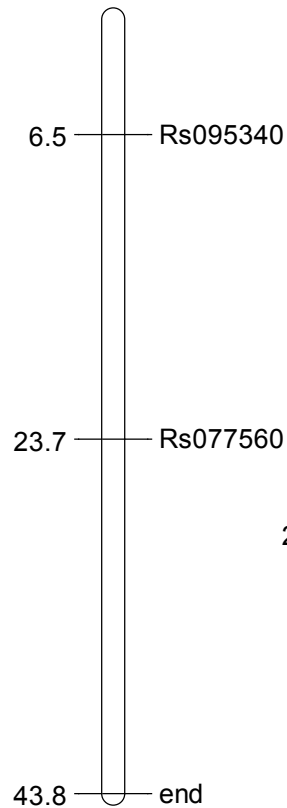**R3**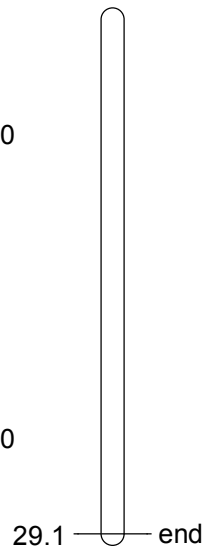**R4**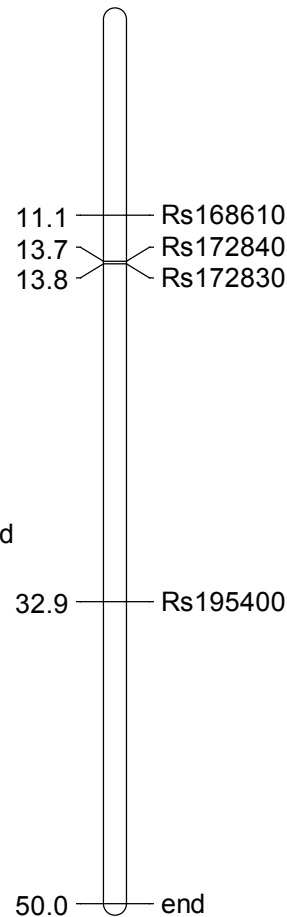**R5**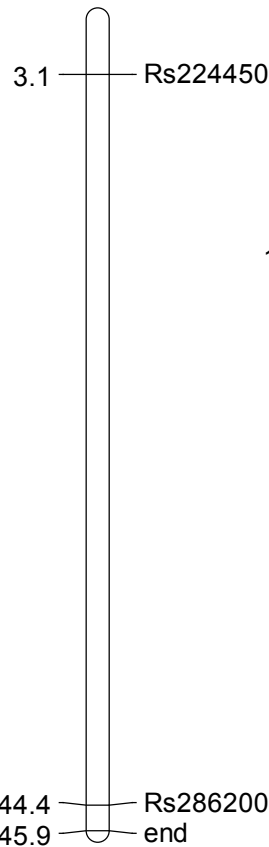**R6**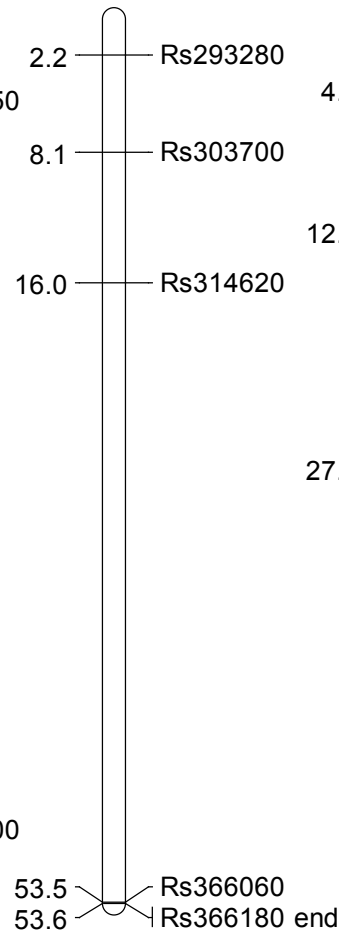**R7**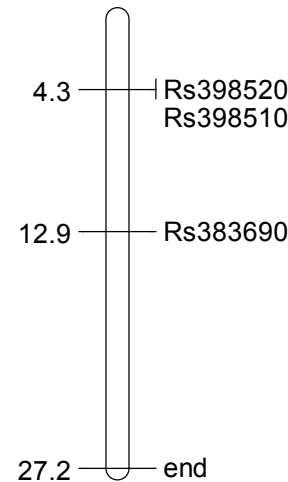**R8**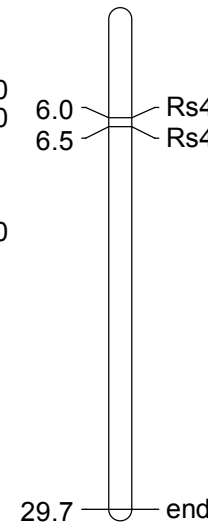**R9**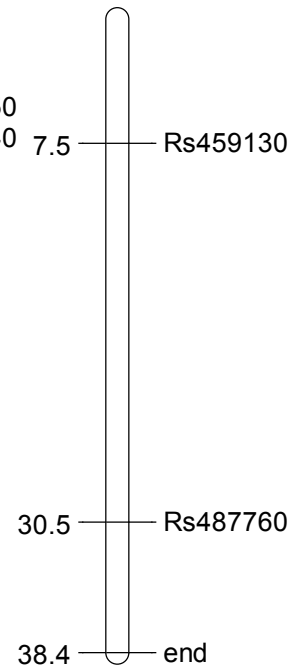

Supplement: S1 Fig — (PDF) [file pone.0204137.s001.pdf]
